# CircNUP54 promotes hepatocellular carcinoma progression via facilitating HuR cytoplasmic export and stabilizing BIRC3 mRNA

**DOI:** 10.1038/s41419-024-06570-4

**Published:** 2024-03-05

**Authors:** Chenwei Tang, Hongkai Zhuang, Wentao Wang, Qingbin Wang, Xiaowu Ma, Bingkun Wang, Ziyu Zhang, Jiahao Jiang, Zhiqin Xie, Wenliang Tan, Lei Yang, Songyao Liu, Yonglin Hua, Yuxin Xiao, Baoshan Ding, Yajin Chen, Changzhen Shang

**Affiliations:** 1 Department of Hepatobiliary Surgery, Sun Yat-sen Memorial Hospital, Sun Yat-sen University, Guangzhou, Guangdong Province 510120 China; 2 Guangdong Provincial Key Laboratory of Malignant Tumor Epigenetics and Gene Regulation, Sun Yat-sen Memorial Hospital, Sun Yat-Sen University, Guangzhou, Guangdong Province 510120 China; 3 Center of Hepatobiliary and Pancreatic Surgery, Zhuzhou Hospital Affiliated to Xiangya School of Medicine, Central South University, Zhuzhou City, Hunan Province 412007 China; 4 Key Laboratory of Stem Cells and Tissue Engineering (Ministry of Education), Zhongshan School of Medicine, Sun Yat-sen University, Guangzhou, China

**Keywords:** Oncogenes, Cancer therapy

## Abstract

Circular RNAs (circRNAs) have been implicated in tumorigenesis and progression of various cancers. However, the underlying mechanisms of circRNAs in hepatocellular carcinoma (HCC) have not been fully elucidated. Herein, a new oncogenic circRNA, hsa_circ_0070039 (circNUP54), was identified to be significantly upregulated in HCC through circRNA sequencing. As verified in 68 HCC samples, circNUP54 overexpression was correlated with aggressive cancerous behaviors and poor outcomes. Moreover, the function experiments showed that knockdown of circNUP54 inhibited the malignant progression of HCC in vitro and in vivo, whereas overexpression of circNUP54 had the opposite role. Mechanistic investigations carried out by RNA pull-down, RNA immunoprecipitation, and immunofluorescence revealed that circNUP54 interacted with the RNA-binding protein Hu-antigen R (HuR) and promoted its cytoplasmic export. The cytoplasmic accumulation of HuR stabilized the downstream BIRC3 mRNA through its binding to the 3′ UTR region. Consequently, the encoded protein of BIRC3, cellular inhibitor of apoptosis 2 (cIAP2), proceeded to activate the NF-κB signal pathway and ultimately contributed to HCC progression. In addition, depletion of BIRC3 rescued the pro-tumorigenic effect of circNUP54 on HCC cells. Overall, this study demonstrated that circNUP54 facilitates HCC progression via regulating the HuR/BIRC3/NF-κB axis, which may serve as a promising therapeutic target for HCC treatment.

## Introduction

Primary liver cancer is the sixth most commonly diagnosed cancer and the third leading cause of cancer-related mortality worldwide [1]. Hepatocellular carcinoma (HCC) is the most common primary liver cancer, accounting for 85–90% of all cases [2]. Owing to the insidious onset of HCC, a substantial proportion of patients are diagnosed at an advanced stage and are unsuitable for radical resection [3]. Despite significant improvements in both surgical and pharmacological treatments, the prognosis of patients with HCC remains poor [4]. Therefore, in-depth investigation into the molecular mechanisms underlying tumorigenesis and progression is essential to uncovering new therapeutic targets for HCC.

Circular RNAs (circRNAs) are a special class of non-coding RNAs characterized by a covalently closed single-stranded circular structure that are produced by back-splicing of exons, introns, or untranslated regions [5, 6]. Aberrant circRNA expression plays a regulatory role in intracellular signal transduction, which contributes to the tumorigenesis of many cancers [7], including breast cancer [8], non-small cell lung cancer [9], and gastric cancer [10]. Generally, most circRNAs exert their functions by sponging microRNAs (miRNAs), which means reducing the repression effect of miRNAs on the targeted mRNA [11]. For instance, circANAPC7 sponges miR-373 to suppress pancreatic cancer [12]. Regarding HCC, many circRNAs, such as hsa_circ_0072309 [13], hsa_circ_0007874 [14], and hsa_circ_0095868 [15], have been identified as miRNA sponges that participate in oncogenesis, progression, and drug resistance of HCC. However, the additional potential mechanisms of action of circRNAs in HCC remain to be elucidated.

RNA-binding protein (RBP), which plays a crucial role in post-transcriptional modification and mRNA translation, has been reported as an important co-worker of circRNA [16]. By binding to specific RBPs, circRNAs act as protein sponges, scaffolds, decoys, or recruiters to influence diverse pathophysiological processes [17]. Several circRNA-related RBPs have been identified in HCC [18–20]. For example, FMRP is competitively bound by circZKSCAN1, leading to a suppression of the binding between FMRP and the oncogene CCAR1 mRNA, which subsequently inhibits the Wnt signaling pathway in HCC [19]. CircIARS promotes the sorafenib-induced ferroptosis of HCC by binding with ALKBH5 [18]. Hu-antigen R (HuR), also known as ELAVL1, is one of the most well-understood RBPs involved in the tumorigenesis of numerous cancers [21]. Accumulating evidence suggests that HuR promotes mRNAs’ stability and translation by binding to U- or AU-rich RNA elements (AREs) in their untranslated regions (UTRs), usually in the 3′ UTR [22, 23], and is regulated by circRNAs [24–26]. Liang et al. reported that circDCUN1D4 interacts with HuR to enhance the binding between HuR and thioredoxin-interacting protein (TXNIP) mRNA, which eventually promotes TXNIP translation [25]. Nevertheless, precisely how HuR is involved in circRNA-regulated HCC progression remains largely unknown.

The present study aimed to explore the unknown roles of circRNAs in HCC. Through the utilization of circRNA sequencing and a series of experimental verifications, a novel carcinogenic circular RNA, circNUP54 (circBase ID: hsa_circ_0070039), was identified in HCC. Clinical correlation analysis, gain and loss of function experiments, RNA pull-down, and RNA immunoprecipitation were conducted to elucidate the role and mechanism of circNUP54. As a result, circNUP54 overexpression indicates a poor prognosis, and circNUP54 knockdown inhibits HCC progression. Mechanistically, circNUP54 interacted with HuR and promoted its cytoplasmic export, further stabilized BIRC3 mRNA, and eventually activated the nuclear factor kappa B (NF-κB) signaling pathway in HCC cells. Overall, our findings provide potential therapeutic targets and novel insights into HCC pathogenesis.

## Materials and methods

### RNA sequencing

The circRNA sequencing was performed to screen the differentially expressed circRNAs in HCC tissues compared with adjacent normal tissues, as previously described by our team [13]. To explore the downstream regulatory mechanisms of circNUP54, transcriptome sequencing followed by bioinformatic analysis was conducted in Huh7 cells with circNUP54 knockdown by the Illumina NovaSeq 6000 platform and NovaSeq 6000 S4 Reagent (San Diego, CA, USA).

### Clinical specimen

Sixty-eight pairs of HCC and adjacent normal tissues were collected from patients undergoing hepatectomy at Sun Yat-sen Memorial Hospital, Sun Yat-Sen University (Guangzhou, China) from 2015 to 2018. Inclusion criteria: (1) Patients with confirmation of HCC by postoperative histology. (2) Patients did not receive targeted medications, transcatheter arterial chemoembolization, chemotherapy, or radiofrequency ablation before surgery. (3) Patients who have never had any other malignancies. (4) Patients with informed consent before the specimen was collected. Exclusion criteria: (1) Patients diagnosed with combined hepatocellular and cholangiocellular carcinomas or cholangiocellular carcinomas. (2) Patients lacking follow-up data or clinical information in full. These samples were primarily used for qRT-PCR verification. Following surgical resection, all samples were immediately snap-frozen and stored in liquid nitrogen. Patient clinicopathological information, including age, cirrhosis, HBV infection, BCLC stage, and grade of histologic differentiation, is presented in Table S1. Informed consent was signed by all patients enrolled in this study.

### Cell culture

Human hepatocarcinoma cell lines, including SUN387, SNU449, HepG2, Li-7, and Huh7, were obtained from the Cell Bank of the Chinese Academy of Sciences (Shanghai, China). Hep3B, PLC/PRF/5, and SMMC-7721 were purchased from ATCC (Manassas, VA, USA). Short Tandem Repeat profiling was used to validate the cell lines, and PCR was used to determine that they were mycoplasma-free. SNU449 and PLC/PRF/5 were grown in RPMI 1640 (Gibco BRL, USA), whereas the remaining cell lines were cultured in DMEM medium (Gibco BRL, USA). All the culture medium was supplemented with 10% fetal bovine serum and 1% penicillin-streptomycin, and the plates were maintained at 37 °C in a humidified atmosphere of 5% CO_2_. The cells were passaged at a 1:3 split ratio when they reached 85–90% confluence.

### Plasmids, siRNA, and lentivirus transfection

The small interfering RNAs (siRNAs) targeting the back-splicing site of circNUP54 and siRNAs for HuR or BIRC3 were designed and chemically synthesized by Guangzhou RiboBio Co., Ltd. A list of siRNA sequences is provided in Table S2. To construct plasmids overexpressing HuR or BIRC3, their cDNA was cloned into the pCDH vectors with technical support provided by Kidan Bio Co., Ltd. Moreover, plasmids expressing wild-type or predicted binding site mutants of circNUP54 were designed by the same laboratory. The Gene Chemical Technology Co., Ltd constructed the lentivirus-circNUP54 and lentivirus-sh-circNUP54, as well as the 3xFLAG-tagged HuR wild-type plasmids and HuR RRM domain-truncated plasmids. As for transfection, cells were seeded in 6-well plates and cultured to a confluence of 60–70%, then transfected with different plamids or siRNAs using Lipo3000 (Invitrogen, Carlsbad, CA, USA). Lentiviral transduction was performed according to the manufacturer’s instructions. Stably transduced cells were selected using puromycin (2 μg/mL).

### RNA extraction and qRT-PCR

Total RNA was extracted from cells or clinical specimens using the EZ-press RNA Purification Kit (EZBioscience, Roseville, USA). The nuclear and cytoplasmic RNAs were isolated and extracted using the NE-PER Kit (Thermo Fisher Scientific, MA, USA). The cDNA was synthesized from 1000 ng of RNA by reverse transcription using the Evo M-MLV RT Kit (Accurate Biotechnology Co., Ltd, Hunan, China) after examining the RNA concentration with Nanodrop (ND-2000) at a rate of 260/280. The final cDNA was used for quantitative real-time polymerase chain reactions (qRT-PCR) using SYBR® Green Pro Taq HS Premix (Accurate Biotechnology Co., Ltd, Hunan, China). GAPDH and U6 were used for internal reference. All the primers were synthesized by Generay Biotechnology (Shanghai, China), and their sequences are presented in Table S2.

### Western blot (WB)

Briefly, cell extracts were prepared using cell lysis buffer for Western and IP (Beyotime, Wuhan, China). The protein concentrations in the supernatant were measured using the bicinchoninic acid test (CWBIO, Beijing, China). The same amounts (20 μg) of total protein were loaded into SDS-PAGE gels (EpiZyme, Shanghai, China) for electrophoresis. After that, separated proteins were electroeluted at 120 volts onto PVDF membranes (Roche Applied Science, Germany). The membranes were blocked with Protein Free Rapid Blocking Buffer (Shanghai Epizyme Biomedical Technology Co., Ltd, China) for 15 min and incubated with primary antibodies (exhibited in Table S3) at a dilution of 1:1000 for 24 h at 4 °C, followed by HRP-conjugated secondary antibodies incubation for 2 h at RT. Finally, bands were visualized using the Omni-ECL^TM^ Femto Light Chemiluminescence Kit (EpiZyme, Shanghai, China) and imaged by the Syngene G: BOX Chemi XT4 system.

### Cell proliferation assay

Cell Counting Kit-8 (CCK-8), colony formation, and 5-Ethynyl-2′-deoxyuridine (EdU) were used for the cell proliferation assay. In brief, transfected cells were seeded in triplicate into 96-well plates (2000 cells per well) and cultured overnight in the same conditions. On days 1–5, CCK8 working solutions (Complete Media: CCK-8 = 10:1) were piped into the wells and incubated in the dark for 3 h. The absorbance (OD450) was detected using a microplate reader (Thermo MK3, Thermo Fisher Scientific, USA). For the colony formation assay, 1 × 10^3^ cells were seeded in a 6-well plate and cultured for two weeks. Cells were fixed with 4% paraformaldehyde fixation and stained with 0.3% crystal violet. For the EdU (Cy3) assay, cells were incubated with Edu working solution (1:1000) for 2 h and then treated with paraformaldehyde for 20 min. After 3 washes in PBS supplemented with 3% BSA, the cells were permeabilized with 0.5% Triton for 15 min, followed by another 3 washes. After being incubated with the click reaction solution for 30 min in the dark, the cells were counterstained with Hoechst for 10 min. The images were captured using a fluorescence microscope (Olympus IX73, Japan).

### Cell migration assay

Transwell and wound healing were conducted for the migration assay. The upper chambers of 24-well transwell inserts (Jet Bio-Filtration Co., Ltd, Guangzhou, China) were plated with 10,000 cells in serum-free medium, and the lower chambers were filled with complete medium supplemented with 20% FBS. The upper chambers were collected after 24 h of culturing, followed by 4% paraformaldehyde fixation for 15 min and 0.3% crystal violet staining for 10 min. Cells that migrated through the pores were counted under the microscope in three random fields. As for the wound healing assay, transfected cells were seeded in 6-well plates and cultured until reaching a confluence of 90–100%. A sterile 1-ml plastic pipette tip was used to make a wound in the cell monolayer. The cells were then grown for 24 h in a medium containing 2% fetal bovine serum after being washed three times in PBS. The gap sizes at 0 and 24 h were measured and recorded by microscope imaging for calculating the migration rate.

### Cell invasion assay

The same transwell inserts were used for the invasion assay. A day before seeding, 200 μl of matrigel (Corning, NY, USA) solution (Matrigel: DMEM = 1:20) was added to each upper chamber to simulate the extracellular matrix. The rest of the steps are the same as described in the transwell migration assay.

### RNase R treatment and Actinomycin D (ActD) assays

For RNase R digestion, 4 μg of total RNA was divided into two groups, followed by incubation with or without 3U/μg RNase R at 37 °C for 30 min. The remaining levels of circNUP54 and NUP54 mRNA were detected by qRT-PCR. The ActD assay was used to verify the stability of circNUP54 and the other mRNAs. SNU449 and Huh7 were incubated with 2 μg/mL ActD (APExBIO, Boston, USA) for 0 h, 4 h, 8 h, 12 h, 16 h, 20 h, and 24 h, respectively. qRT-PCR was used to determine the remaining relative circRNA and mRNA, as well as the stability of BIRC3 mRNA.

### Fluorescent in situ hybridization (FISH)

The cy3-labeled fluorescent probes targeting circNUP54 and 18 S were synthesized by GenePharma (Shanghai, China), and their sequences are listed in Table S2. Briefly, HUH7 and SNU449 cells (2 × 10^3^) were seeded in confocal dishes, fixed with 4% paraformaldehyde, and permeabilized with 0.4% Triton X-100. Then the cells were hybridized with labeled probes in the reaction buffer over night at 37 °C. Subsequently, the cell nucleus was stained with DAPI and photographed by confocal microscopy. 18 S was used as a positive control for the cytoplasmic fraction.

### CircRNA pull-down and mass spectrometry (MS)

The RNA antisense purification (RAP) kit (BersinBio, Guangzhou, China) was used for the circRNA pull-down assay. The biotin-labeled probe to the back-splicing site of circNUP54 and the negative control (NC) probe were synthesized by Gene Pharma (Gene Pharma, Suzhou, China). Table S2 lists the sequences of probes. Briefly, a cell lysate harvested from circNUP54-overexpressing cells (1 × 10^7^) was treated with DNase, followed by separately incubating with each probe (circNUP54 and NC) at 70 °C for 30 min. Subsequently, streptavidin magnetic beads were added to the RNA mixture and then hybridized with probes at room temperature for 1 h. Next, the magnetic beads were divided into two portions to purify the pulled-down protein or RNA, respectively. Finally, the purified proteins were subjected to MS (Bioprofile, Shanghai, China) or western blotting, and the RNA was used for RT-qPCR analysis.

### RNA immunoprecipitation (RIP)

The RNA Immunoprecipitation (RIP) Kit (BersinBio, Guangzhou, China) was used to verify the binding RNA to HuR using an anti-HuR antibody. In brief, Huh7 and SNU449 (2 × 10^7^) were collected and lysed. Equal amounts of antibodies (anti-HuR, anti-Ago2, and IgG) were added to a reaction buffer and immobilized on magnetic beads via incubation at 37 °C for 1 h. Subsequently, the antibody-conjugated beads were mixed with cell lysates before incubation overnight in a vertical mixer at 4 °C. The beads were washed four times, then treated with proteinase K for 40 min at 65 °C. Lastly, the corprecipitated RNA was purified and analyzed by RT-qPCR. The antibody information is presented in Table S3.

### Immunohistochemistry (IHC) and hematoxylin-eosin (HE) staining

Following paraformaldehyde fixation for more than 24 h, samples were embedded in paraffin. For IHC, 5-µm-thick sections were deparaffinized in xylene and incubated with 3% H_2_O_2_ for 10 min. A microwave thermal repair method was used to retrieve antigens. The sections were then incubated with the primary antibodies (1:100 dilution) overnight at 4 °C. After being incubated with secondary antibodies for 30 min at room temperature, the sections were revealed using DAB and counterstained with hematoxylin. At last, the slides were scanned to generate analyzable images. HE staining was performed using a hematoxylin and eosin staining kit (Beyotime, Shanghai, China).

### Confocal immunofluorescence (IF) assay

The confocal IF assay was used for localization analysis of circRNA or HuR. The SNU449 and Huh7 cells seeded in confocal dishes were fixed and permeabilized. Cells were blocked for 2 h and then reacted with the primary antibody overnight and the secondary antibody for 2 h. After staining with DAPI, cells were subjected to image acquisition by a confocal microscope.

### Animal studies

Stable transfected cell lines were established and were used to construct subcutaneous and orthotopic xenograft models. HuH7 (Lv-vector, Lv-sh-circ) and Hep3B (Lv-vector, Lv-oe-circ) were used to assess the effect on tumor phenotype after knockdown or overexpression of circNUP54, respectively.

On the basis of the premise that notable differences could be found between groups, the sample size for each was chosen. The 24 BALB/c nude mice (5 weeks old) were divided into four groups at random. A total of 7 × 10^6^ cells suspended in a 150 μL PBS-Matrigel mixture (PBS: Matrigel = 2:1) were injected subcutaneously into the posterior neck of the mice. The tumor volume (length × width^2^ × 0.5) was measured and recorded every 5 or 6 days in a blinded manner. The mice were sacrificed 30 days later, or when the tumor volume reached 1500 mm^3^, and the tumors were isolated, weighed, and subjected to IHC staining. As for the orthotopic xenograft model, 7 × 10^6^ cells per group were inoculated subcutaneously into the posterior neck of BALB/c nude mice. Once tumors reached 1 cm in diameter, they were isolated and cut into small pieces (1 mm^3^). The small tumor masses were then surgically inoculated into the right liver parenchyma of the mice (4 mice per group), aged 5 weeks. A Xenogen IVIS Spectrum Imaging System (Xenogen, CA, USA) was used to detect the tumor bioluminescence intensity weekly in a blinded manner. Mice were sacrificed after 4 weeks, and their livers were subjected to ex-vivo fluorescence imaging. Finally, the samples were fixed in 4% paraformaldehyde, paraffin embedded, and used for HE or IHC staining. In lung metastasis models, the mice received a tail vein injection of 1 × 10^6^ cells suspended in 150 μL PBS and were sacrificed directly after breeding for one mouth. The lungs were harvested for HE staining. In all cases, the Animal Ethics Committee of Sun Yat-sen University approved the animal experiments, which were conducted in the Animal Center of the university.

### Statistical analysis

All data were presented as mean ± standard deviation (SD) from triplicated runs and analyzed using SPSS 21.0 (Version 21.0, SPSS Inc., Chicago, IL, USA). Comparisons were performed using the Student’s *t* test, two-way analysis of variance, or Chi-square test, as appropriate. Survival curves were drawn using the Kaplan-Meier univariate survival analysis and the log-rank test. The Cox proportional hazards model was used for multivariate analysis with a 95% confidence interval. Statistical significance was set at *p* < 0.05. The GraphPad Prism (GraphPad Prism version 8.0; San Diego, CA, USA) was used for the results demonstration. Image J (Image J Software, National Institutes of Health, Bethesda, MD, USA) was used for wound area determination, cell counting, and band density measurement. The Supplementary Material provided the original images of three independent WB experiments.

## Results

### CircNUP54 is upregulated in HCC tissues and is predictive of a poor prognosis

**Fig. 1:**
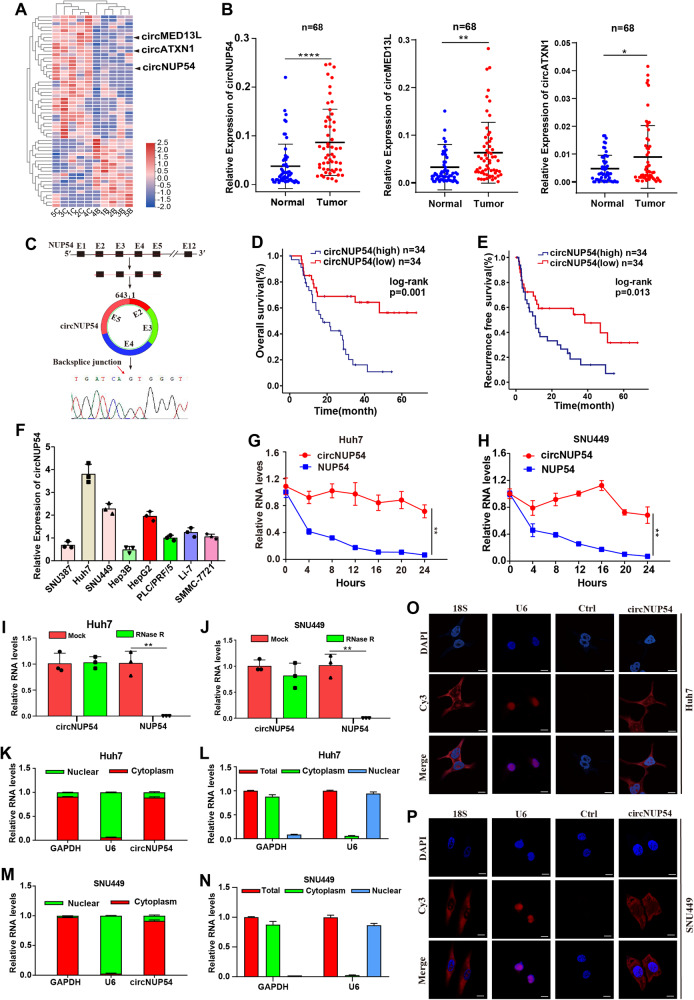
Identification of the upregulated circNUP54 and its characteristics in HCC. **A** Clustered heat map of differentially expressed circular RNAs in five HCC and adjacent non-cancer liver tissues. The top three highly expressed circRNAs (circMED13L, circNUP54, and circATXN1) are labeled. **B** qRT-PCR analysis from 68 pairs of HCC and adjacent tissues shows that circNUP54 was the most significantly overexpressed circRNA. **C** CircNUP54 was backspliced by exons 2, 3, 4, and 5 of its host gene NUP54, and Sanger sequencing validated the back-splice site. **D**, **E** Survival analysis reveals the negative correlation between circNUP54 expression and OS or RFS in patients with HCC. **F** Expression levels of circNUP54 in eight HCC cell lines. **G**, **H** qRT-PCR detection of circNUP54 and NUP54 mRNA after ActD treatment in Huh7 and SNU449 cells, indicating the higher stability of circNUP54. **I**, **J** qRT-PCR analysis to confirm the resistance of circNUP54 to RNase R in Huh7 and SNU449 cells. **K**–**N** qRT-PCR assays after nucleocytoplasmic separation show a mostly cytosolic distribution of circNUP54, with U6 and GAPDH as references for the nucleus and cytoplasm in Huh7 and SNU449 cells. **O**, **P** FISH results demonstrated that circNUP54 is mainly located in the cytoplasm. The 18S and U6 were used as positive controls for cytosolic and nuclear fractions, respectively. Scale bar = 10 μm. Data presented as means ± SD of three independent experiments. **p < 0.01, ****p < 0.0001 (Student’s *t*-test).

Differentially expressed circRNAs were identified via high-throughput sequencing of five paired HCC and normal samples. The three highest-ranked expression circRNAs (hsa_circ_0028587, hsa_circ_0070039, and hsa_circ_0007909, termed circMED13L, circNUP54, and circATXN1, respectively) were selected for further analysis (Fig. 1A) and validated as circRNAs through circBase (http://www.circbase.org/) (Fig. S1A). Initially, divergent primers were designed and used for qRT-PCR verification of 68 paired clinical HCC samples. We found that these three circRNAs were significantly upregulated in HCC tissues, and circNUP54 showed the most pronounced difference in expression (Fig. 1B). Thus, circNUP54 was selected for further analysis. The qRT-PCR product amplified by the circNUP54 primer was subjected to Sanger sequencing, confirming the back-splice site is consistent with circBase annotation. CircNUP54 was generated from two to five exons of the NUP54 gene via back-splicing, with a length of 643 nucleotides (Fig. 1C). Then, the clinical correlation analyses suggest that high levels of circNUP54 contributed to unfavorable overall survival (OS) and recurrence-free survival (RFS) (Fig. 1D, E). High circNUP54 expression is correlated with advanced TNM stage (AJCC classification), higher levels of alpha-fetoprotein (AFP), larger tumor size, and worse tumor differentiation (Table S4). Univariate and multivariate Cox regression analyses showed that TNM stage and positive microvascular invasion were independent prognostic risk factors for OS and RFS, respectively (Tables S5, 6). Finally, circNUP54 expression was analyzed in eight human hepatocarcinoma cell lines (Fig. 1F). Given the high levels of circNUP54, SNU449 and Huh7 cell lines were selected as candidates for follow-up studies. The half-life of circNUP54 was significantly prolonged compared with that of linear NUP54 mRNA after ActD treatment (Fig. 1G, H). Treatment with RNase R also revealed that circNUP54 was more resistant to enzymatic digestion than linear NUP54 mRNA (Fig. 1I, J). The FISH coupled with cytosolic/nuclear fractionation experiments demonstrated that circNUP54 was mainly located in the cytoplasm (Fig. 1K–P). Taken together, these findings demonstrate the circularity of circNUP54, which is abnormally upregulated in HCC and is correlated with poor outcomes.

### CircNUP54 promotes the proliferation, migration, and invasion of HCC cells in vitro

**Fig. 2:**
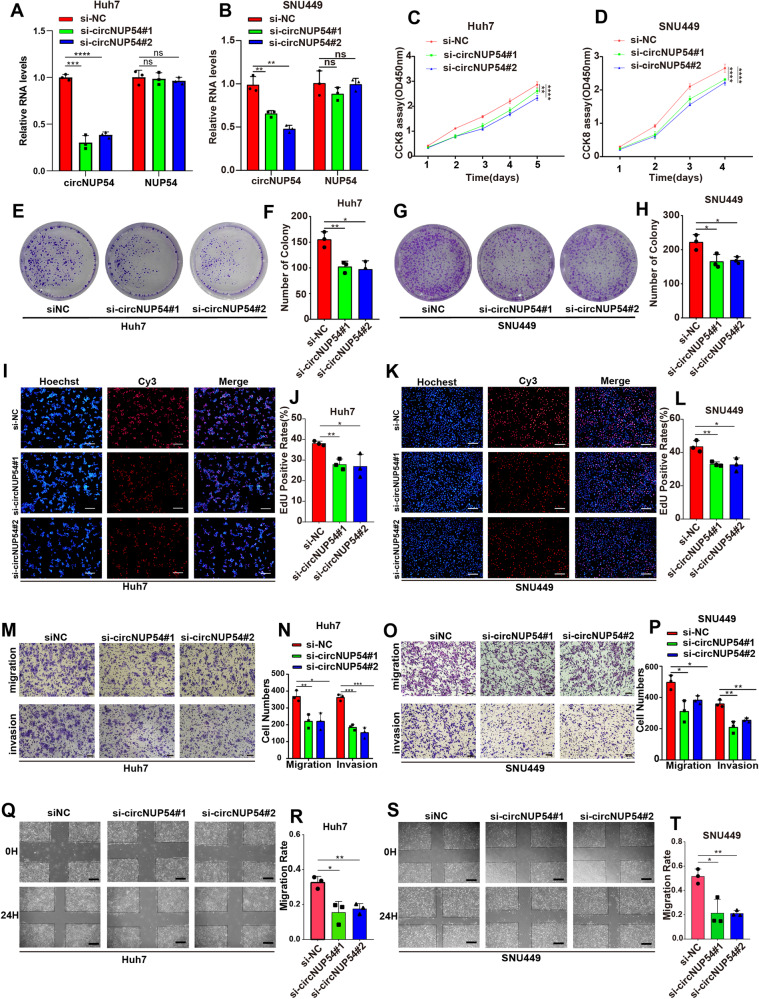
CircNUP54 knockdown inhibits HCC cell proliferation, migration, and invasion in vitro. **A**, **B** qRT-PCR analysis validated the successful knockdown of circNUP54, but not that of NUP54 mRNA, in Huh7 and SNU449 cells transfected with back-splice junction-specific siRNAs. **C**, **D** Growth curves based on CCK-8 assays show the proliferation-inhibitory effect of circNUP54 knockdown on Huh7 and SNU449 cells. **E**–**H** Effect of circNUP54 silencing on HCC cells evaluated using colony formation assay. **I**–**L** The EdU assays of indicated Huh7 and SNU449 cells. Scale bar = 100 μm. **M**–**P** Transwell assays show that circNUP54 knockdown attenuated the cell migration and Matrigel invasion abilities of Huh7 and SNU449 cells. Scale bar = 100 μm. **Q**–**T** Cell migratory ability was decreased in circNUP54-downregulated HCC cells, as detected using the wound-healing assay. Scale bar = 200 μm. Data presented as means ± SD of three independent experiments. *p < 0.05, **p < 0.01, ***p < 0.001, ****p < 0.0001 (Student’s *t*-test).

To investigate the role of circNUP54 in HCC cells, two junction-specific siRNAs (Fig. S1B) of circNUP54 were synthesized and transfected into Huh7 and SNU449 cells. The siRNAs significantly downregulated circNUP54; however, linear NUP54 mRNA expression was not altered (Fig. 2A, B). CCK-8 and colony formation assays revealed that silencing of circNUP54 notably impeded cell proliferation in Huh7 and SNU449 cells compared with that in the control (Fig. 2C–H). A similar pro-proliferative effect of circNUP54 was observed in EdU assays (Fig. 2I–L). Moreover, cell migration and invasion abilities were markedly inhibited when circNUP54 was knocked down, as verified using Transwell assays (Fig. 2M–P). Wound-healing assays also demonstrated that circNUP54 downregulation weakened cell mobility (Fig. 2Q–T). Hep3B cells were chosen for high-expression studies because they expressed circNUP54 at relatively low levels (Fig. 1G). After verifying circNUP54 overexpression via qRT-PCR (Fig. S1C), the Hep3B cells were subjected to in vitro experiments. CCK8, EdU, and colony formation assays showed that the circNUP54-overexpressing group (Lv-circNUP54) proliferated faster than the control group (Lv-NC) (Fig. S1D–H). Transwell and wound healing assays showed that circNUP54 overexpression increased the migration and invasion rates of Hep3B cells (Fig. S1I–N). Collectively, these findings suggest that circNUP54 plays an oncogenic role in HCC cells in vitro.

### CircNUP54 facilitates HCC growth and metastasis in vivo

**Fig. 3:**
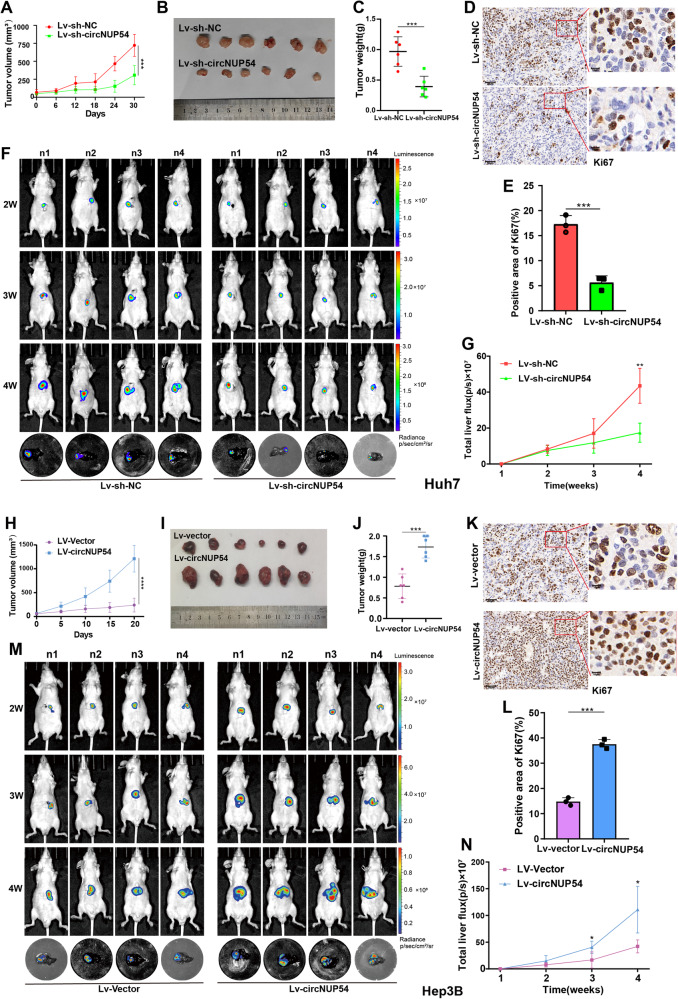
CircNUP54 facilitates HCC growth in vivo. **A** Growth curves of subcutaneous tumors in nude mice (*n* = 6). Lv-sh-NC and Lv-sh-circNUP54 refer to Huh7 cells with negative control and stable circNUP54 knockdown, respectively. **B**, **C** Outlook and weight of subcutaneous xenografts dissected from the mice at the endpoint. **D**, **E** Representative IHC images of KI67 staining of subcutaneous tumors and the results of quantitative analysis. Left scale bar = 50 μm, right scale bar = 10 μm. **F** IVIS images of orthotopic xenograft tumor growth from each mouse and ex vivo fluorescence images of isolated livers collected at the endpoint (n = 4). **G** Quantitative analysis of the liver fluorescence intensity over time. **H**–**N** Same type illustrations with **A**–**G**, based on Hep3B cells stably transfected with the vector (Lv-vector) or circNUP54 (Lv-circNUP54). Both subcutaneous and orthotopic xenograft tumor models in nude mice suggested that circNUP54 overexpression promoted Hep3B cell growth in vivo. Data presented as means ± SD of three independent experiments. *p < 0.05, ***p < 0.001 (Student’s *t*-test).

To further evaluate the biological function of circNUP54 in vivo, Huh7 cells with stable circNUP54 knockdown and Hep3B cells with stable circNUP54 overexpression were used to establish subcutaneous tumor models. Knockdown of circNUP54 (Lv-sh-circNUP54) significantly decreased the tumor growth rate compared with that in the control group (Lv-sh-NC) in Huh7 cells (Fig. 3A–C, Fig. S2A). Conversely, circNUP54 overexpression (Lv-circNUP54) resulted in a higher tumor growth rate than the control group (Lv-vector) in Hep3B cells (Fig. 3H–J, Fig. S2B). Fig. S2C, D verified the change of circNUP54 level in the subcutaneous tumors. IHC staining revealed that the level of Ki67 decreased after circNUP54 knockdown (Fig. 3D, E), whereas circNUP54 upregulation exhibited the opposite trend (Fig. 3K, L). In addition, an orthotopic xenograft model (a more convincing animal model) was employed to investigate the effects of circNUP54 on tumor growth (Fig. 3F, M). The growth rate of fluorescence intensity was impeded with increasing age in the Lv-sh-circNUP54 group compared with that in the Lv-sh-NC group (Fig. 3G). The same pro-fluorescence intensity-increasing effect of circNUP54 was observed in Hep3B cells (Fig. 3N). Interestingly, HE staining of orthotopic xenografts indicated that tumor margins were more tortuous in the circNUP54-upregulated groups, indicating a more aggressive cancerous feature (Fig. S2E). Moreover, lung metastasis models were established using Huh7 cells with stable circNUP54 overexpression or knockdown. CircNUP54 knockdown dramatically decreased the number and size of metastatic lesions, whereas circNUP54 overexpression aggravated lung metastatic lesions (Fig. S2F–H). These findings suggest that circNUP54 facilitates HCC cell proliferation and metastasis in vivo.

### CircNUP54 knockdown inactivates the NF-κB pathway via downregulation of BIRC3

**Fig. 4:**
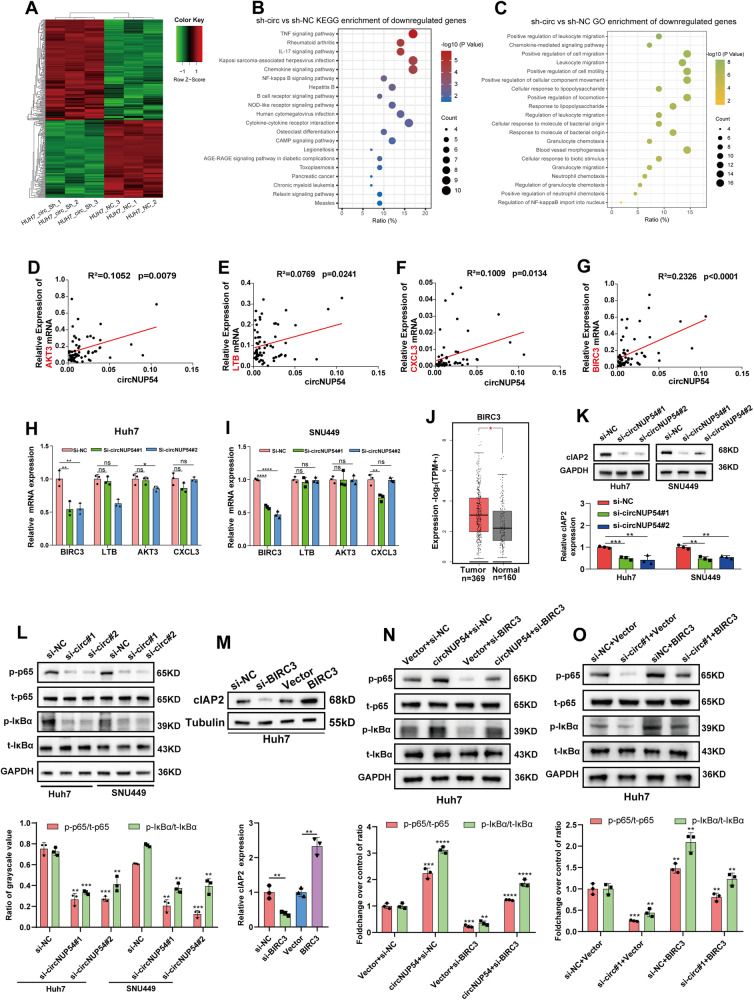
CircNUP54 knockdown inactivates the NF-κB pathway via downregulation of BIRC3. **A** Clustered heat map of RNA transcriptome sequencing in Huh7 cells transfected with sh-NC or sh-circNUP54. **B**, **C** KEGG and GO enrichment analyses of downregulated genes after circNUP54 knockdown. **D**–**G** qRT-PCR of 68 HCC samples verified the correlation of circNUP54 with four downstream target genes, selected by overlapping the top 20 most significantly downregulated genes and NF-κB pathway-involved genes. **H**, **I** qRT-PCR was performed to determine the expression of BIRC3, LTB, AKT3, and CXCL3 in Huh7 and SNU449 cells with circNUP54 knockdown. **J** BIRC3 expression in HCC and normal liver tissue (TCGA database). **K** WB analysis of cIAP2, a protein encoded by BIRC3 mRNA, in Huh7 and SNU449 cells with circNUP54 knockdown. GAPDH served as an internal reference. **L** WB and quantitative analysis of the NF-κB pathway-related proteins (p-p65, p65, IκBα, and p-IκBα) in indicated HCC cells after circNUP54 knockdown. **M** The cIAP2 expression of Huh7 cells after transfection with siRNAs (si-NC and si-BIRC3) or plasmids (Vector or BIRC3). Tubulin was used as an internal control. **N** WB and quantification results of p-p65/t-p65 and p-IκBα/t-IκBα in Huh7 cells, showing that knockdown of BIRC3 impaired the activation of the NF-κB pathway caused by circNUP54 overexpression. **O** Overexpression of BIRC3 rescued the inhibitory effect of circNUP54 knockdown on the NF-κB pathway in Huh7 cells. Data presented as means ± SD of three independent experiments. *p < 0.05, **p < 0.01 (Student’s t-test).

To explore the downstream regulatory mechanisms of circNUP54-mediated oncogenic effects in HCC cells, RNA transcriptome sequencing (RNA-seq) was performed on Huh7 cells after circNUP54 knockdown (Fig. 4A). The Kyoto Encyclopedia of Genes and Genomes (KEGG) pathway enrichment and Gene Ontology (GO) analysis showed that the tumor necrosis factor α (TNF-α) and NF-κB pathways, two overlapping and closely related axes, were significantly enriched (Fig. 4B, C, Fig. S3A, B). By intersecting the top 20 most significantly downregulated genes and TNF-α or NF-κB pathway-involved genes, the four candidate genes (ATK3, LTB, CXCL3, and BIRC3) were selected for further verification. First, their correlation of expression with circNUP54 levels were analyzed in 68 HCC samples. All four genes were significantly positively correlated with circNUP54, which was consistent with the RNA-seq results, with BIRC3 being the most significantly correlated gene (R^2^ = 0.2326, *p* < 0.0001) (Fig. 4D–G). In addition, BIRC3 mRNA expression was markedly reduced after silencing circNUP54 compared with that in the other three genes (Fig. 4H, I). Analysis of the TCGA database revealed a significant upregulation of BIRC3 in HCC tissues (Fig. 4J, Fig. S3C). WB also revealed that the protein encoded by BIRC3, cellular inhibitor of apoptosis 2 (cIAP2), was decreased in circNUP54-interfered cells (Fig. 4K). BIRC3/cIAP2 was abnormally elevated in the majority of human malignancies and promoted tumor progression by activating the NF-κB pathway [27–29]. Thus, the key proteins of the NF-κB pathway were determined, and the results demonstrated that the ratio of phosphorylated and total p65 (p-p65/t-p65) as well as phosphorylated and total IκBα (p-IκBα/t-IκBα) had decreased following the silencing of circNUP54 in both Huh7 and SNU449 cells (Fig. 4L). An overexpression plasmid and a siRNA targeting BIRC3 were constructed and successfully changed the levels of BIRC3 (Fig. 4M, Fig. S3D–F). The activated or inactivated effect on the NF-κB pathway caused by circNUP54 could be rescued by knockdown or overexpression of BIRC3, respectively, indicating that BIRC3 is the downstream target of circNUP54 (Fig. 4N, O). In summary, the above findings imply that circNUP54 knockdown inactivates the NF-κB pathway via downregulation of BIRC3.

### CircNUP54 interacts with the RBP HuR in HCC

**Fig. 5:**
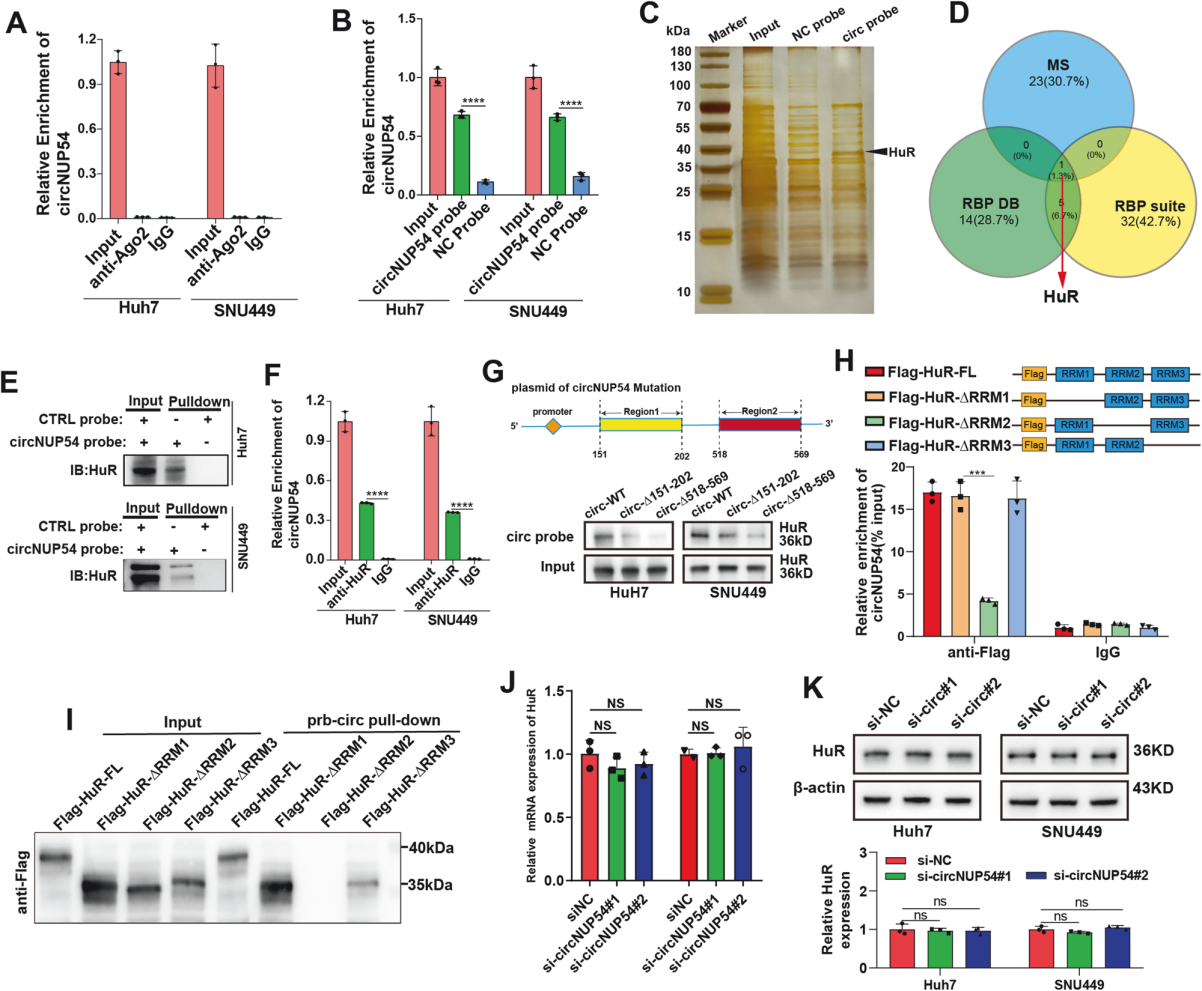
CircNUP54 interacts with the RBP HuR in HCC. **A** RIP-qPCR analysis showed that circNUP54 was not enriched with the AGO2 antibody. **B** qRT-PCR results of RNA pull-down products using a biotin-labeled circNUP54 probe and control probe in circNUP54-overexpressing HCC cells. **C** Silver staining of circNUP54 pull-downs. **D** HuR was found to interact with circNUP54 by overlapping MS and target RBPs predicted using the RBP suite and RPBDP databases. **E** WB results verifying HuR enrichment using the circNUP54 probe. **F** RIP-qPCR showing precipitation of circNUP54 with anti-HuR. **G** Schematic representation of circNUP54 overexpression plasmids with predicted binding regions truncated (top panel). WB was used to detect HuR pulled-down by the circNUP54 probe in the lysates of Huh7 and SNU449 cells after transfection with wild-type or truncated circNUP54 overexpression plasmids (bottom panel). **H** Plasmids expressing FLAG-labeled FL or RRM-truncated HuR were transfected into circNUP54-overexpressing Huh7 cells, and then the RIP products pulled by anti-FLAG were analyzed by qRT-PCR to evaluate the enrichment efficiency of circNUP54. **I** 293T cells transfected with FL or RRM-truncated HuR plasmids were subjected to RNA pull-down using a circNUP54 probe. WB detected the pulled-down HuR by anti-FLAG. **J**, **K** HuR expression remained unchanged, as detected using RT-qPCR and WB after silencing circNUP54. Data presented as means ± SD of three independent experiments. ***p < 0.001, ****p < 0.0001 (Student’s *t*-test).

Next, we explored the mechanism by which circNUP54 promotes BIRC3 expression. Given that “miRNA sponges” have been extensively explored as a mechanism in circRNA studies [30–32], we initially investigated this potential mechanism. The RIP assays using anti-argonaute 2 (anti-AGO2) showed that there were no significant enrichments of circNUP54 in the AGO2 group compared with the IgG group, indicating that circNUP54 may not act as a “miRNA sponge” (Fig. 5A). Since interactions with RBPs are important mechanisms by which circRNAs regulate downstream genes, a new direction has been established. RNA pull-down assays using the biotin-labeled circNUP54 probe showed that circNUP54 was successfully enriched compared with the control probe (Fig. 5B), indicating good specificity of the probe. The pulled-down proteins were subjected to SDS-PAGE and MS (Fig. 5C). The online databases RBPDB (http://rbpdb.ccbr.utoronto.ca/index.php) and RBP Suite (http://www.csbio.sjtu.edu.cn/bioinf/RBPsuite/) were used to screen for the potential RBPs of circNUP54. We found that only RBP-HuR overlapped in the MS and database screening (Fig. 5D). The HuR was significantly pulled down by the circNUP54 probe in Huh7 and SNU449 cells (Fig. 5E), and the circNUP54 was also precipitated using an anti-HuR antibody in the RIP assay (Fig. 5F). According to CatRAPID (http://service.tartaglialab.com) prediction, the two most likely binding sites of circNUP54 were located at 151–202 nt and 518–569 nt (Fig. S4A). Subsequently, we constructed circNUP54 plasmids with these regions truncated and transfected them into Huh7 and SNU449 cells. The RNA pull-down assays suggested that both 151–202 nt and 518–569 nt region truncations of circNUP54 induced a marked reduction of circNUP54-captured HuR compared with that of the WT, with a more pronounced reduction in the 518-569 nt region, indicating that region 518-569 is the more critical binding site of circNUP54 that interacts with HuR (Fig. 5G).

HuR contains three RNA recognition motifs (RRMs) and a nucleocytoplasmic shuttling (HNS) sequence [33, 34]. To determine which RRM is responsible for its interaction with circNUP54, FLAG-tagged plasmids encoding full-length (FL) or truncated HuR (∆RRM1, ∆RRM2, or ∆RRM3) were constructed and transfected into circNUP54-overexpressing Huh7 cells. RIP assays, precipitated with anti-FLAG, demonstrated that the circNUP54 could not be enriched by the RRM2-truncated HuR (Fig. 5H). Lysates of 293T cells transfected with HuR mutant plasmids were co-incubated with a biotin-labeled circNUP54 probe to conduct the pull-down assay. The RRM2-truncated HuR could not be pulled down by the circNUP54 probe, indicating that circNUP54 mainly interacted with the RRM2 of HuR (Fig. 4I). According to CatRAPID, the most likely position for HuR binding to circNUP54 is located at positions 126–177, which are contained in RRM2 (Fig. S4B). This prediction was confirmed by the experimental results. Finally, the qRT-PCR and WB revealed that circNUP54 did not alter HuR expression when bound to it (Fig. 5J, K). Thus, circNUP54 interacts with HuR by binding to RRM2.

### HuR stabilizes BIRC3 mRNA depending on circNUP54-induced cytoplasmic export

**Fig. 6:**
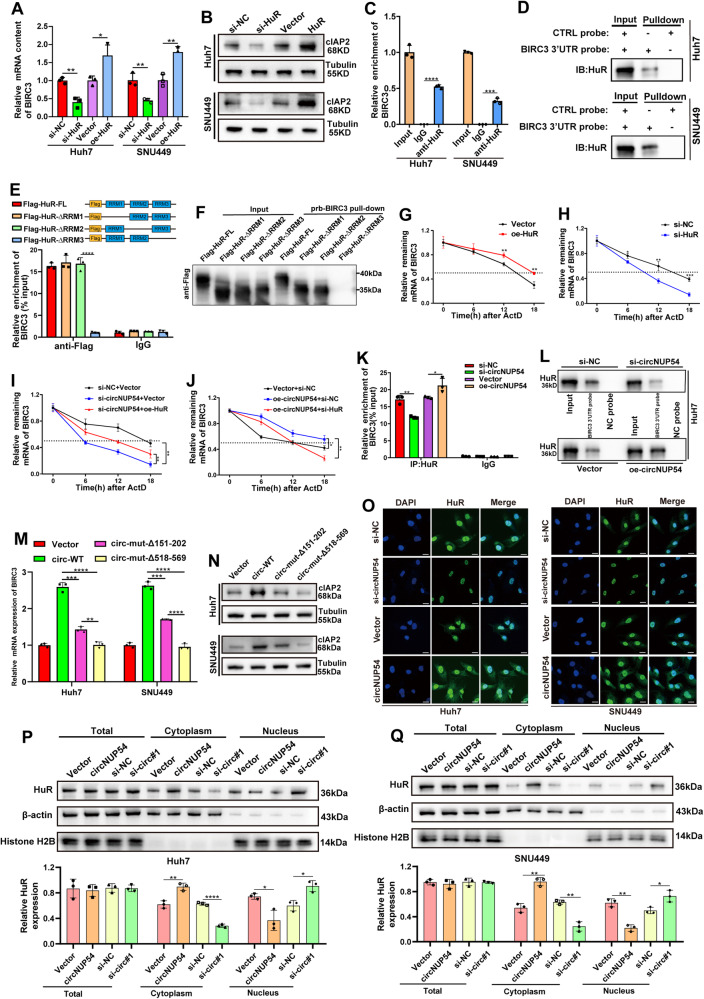
HuR stabilizes BIRC3 mRNA depending on circNUP54-induced cytoplasmic export. **A**, **B** BIRC3 mRNA and its encoded protein, cIAP2, were detected using RT-qPCR and WB in transient HuR knockdown or overexpression HCC cells, respectively. **C** RIP assay indicated BIRC3 mRNA was enriched by HuR. **D** RNA pull-down assay revealed HuR was pulled-down by the BIRC3 3’UTR probe. **E** Plasmids expressing FLAG-labeled FL or RRM-truncated HuR were transfected into Huh7 cells. The RIP products, precipitated using anti-FLAG, were subjected to qRT-PCR analysis to assess BIRC3 mRNA enrichment. **F** The 293T cells transfected with FL or RRM-truncated HuR plasmids were subjected to RNA pull-down using a BIRC3 3′UTR probe. WB detected the pulled-down HuR by anti-FLAG. **G**, **H** The remaining BIRC3 mRNA was detected using qRT-PCR in HuR up-regulated or down-regulated Huh7 cells after ActD treatment. **I**, **J** Degradation rate of BIRC3 mRNA in Huh7 cells transfected with indicated siRNAs or plasmids. **K** CircNUP54 overexpression or knockdown Huh7 cells were subjected to RIP assays precipitated by HuR. The enrichment of BIRC3 mRNA was analyzed using qRT-PCR. **L** BIRC3 3’UTR probe pull-down was performed in Huh7 cells with circNUP54 overexpression or knockdown. The pulled down HuR was detected using WB. **M**, **N** BIRC3 mRNA and cIAP2 were determined using RT-qPCR and WB in HCC cells transfected with wild-type or mutant plasmids of circNUP54. **O** IF images of HuR (green) in circNUP54 knockdown or overexpression HCC cells. CircNUP54 overexpression promotes the cytosolic accumulation of HuR; Scale bar = 10 μm. **P**, **Q** Quantitative WB determining the HuR levels in total lysates or subcellular fractions of the circNUP54 overexpression or knockdown HCC cells. Cytosolic HuR was increased by circNUP54 overexpression. Data presented as means ± SD of three independent experiments. *p < 0.05, **p < 0.01, ***p < 0.001 (Student’s *t*-test).

Because HuR played a role in post-transcription regulation by stabilizing target mRNA [22, 23], we hypothesized that HuR may affect the expression of the circNUP54 downstream gene, BIRC3, by stabilizing its mRNA. Initially, we found that HuR knockdown was followed by a decrease in BIRC3 mRNA and protein levels, whereas HuR overexpression increased BIRC3 expression (Fig. 6A, B; Fig. S5A–D). In the RIP assay, anti-HuR significantly enriched BIRC3 mRNA compared with IgG (Fig. 6C). The RBPmap predicted HuR-binding sites in the BIRC3 3′ UTR (Fig. S5E, Supplementary Excel), and then a biotin-labeled 3′ UTR mimic probe identical to the binding site sequence was synthesized. RNA pull-down assay revealed that HuR could be pulled-down by the 3′ UTR probe of BIRC3 mRNA (Fig. 6D), indicating that HuR binds to BIRC3. To clarify which HuR RRM binds to BIRC3 mRNA, FL and RRM-truncated HuR plasmids (∆RRM1, ∆RRM2, or ∆RRM3) were transfected into Huh7 cells. RIP analysis revealed that BIRC3 mRNA was not enriched when RRM3 was truncated (Fig. 6E). Additionally, 293T cells transfected with HuR mutant plasmids were subjected to RNA pull-down using a biotin-labeled BIRC3 3′ UTR probe. It turns out that RRM3-truncated HuR could not be pulled-down by the BIRC3 probe (Fig. 6F), implying that RRM3 is the binding site for HuR-BIRC3 mRNA interaction. In addition, ActD assays showed that HuR overexpression increased the level of BIRC3 mRNA and prolonged its half-life (Fig. 6G), whereas HuR knockdown had the opposite trend (Fig. 6H). HuR and BIRC3 expression showed a significant positive correlation, according to the TCGA database (Fig. S5F).

To explore the role of circNUP54 in regulating the interaction between HuR and BIRC3, rescue experiments of ActD assays were performed. As shown in Fig. 6I, HuR overexpression rescued the circNUP54 knockdown-induced reduction in BIRC3 expression. HuR knockdown reversed the increase in BIRC3 expression caused by circNUP54 overexpression (Fig. 6J). Consistently, circNUP54 knockdown decreased the binding of BIRC3 mRNA and HuR, whereas circNUP54 overexpression increased the binding of them, as verified by the RIP assays (Fig. 6K). Pulldown assays indicated that silencing or upregulation of circNUP54 decreased or increased the enrichment of HuR pulled down by BIRC3 mRNA, respectively (Fig. 6L, Fig. S5G). Additionally, circNUP54 plasmids with mutant HuR-binding sites (∆151–202 or ∆518–569) were transfected into Huh7 and SNU449 cells. The qRT-PCR and WB analysis showed that both circ-mut-∆518–569 and circ-mut-∆151–202 groups exhibited lower BIRC3 expression compared with circ-WT group, but a superior reversion effect on BIRC3 expression was found in the circ-mut-∆518–569 group (Fig. 6M, N, Fig. S5H, I). As a nucleoplasmic shuttle protein, HuR is not only overexpressed but also “overactive” in cancer tissues, characterized by increased subcellular localization in the cytoplasm, which is believed to be essential for HuR-mediated mRNA stabilization [23]. Here, we found that the export of HuR from the nucleus to the cytoplasm could be induced by circNUP54 overexpression, whereas circNUP54 knockdown decreased the cytoplasmic accumulations of HuR, as verified in both IF and WB (Fig. 6O–Q). Fig. S5J showed the motif of HuR. In addition, overexpression of circNUP54 with region 518–569 or 151–202 mutants no longer promoted tumor growth compared with WT in vivo, indicating that both region 518-569 and 151–202 played roles in the function of circNUP54 (Fig. S5K–N). The reason that no significant difference was caught between the LV-circ-mut-∆518–569 and LV-circ-mut-∆151–202 groups may be the insufficient sample size (n = 5). In summary, our findings suggest that circNUP54 acts as a decoy for HuR to promote its cytoplasmic accumulation, thereby stabilizing BIRC3 mRNA.

### CircNUP54 promotes HCC progression by targeting BIRC3 via HuR

**Fig. 7:**
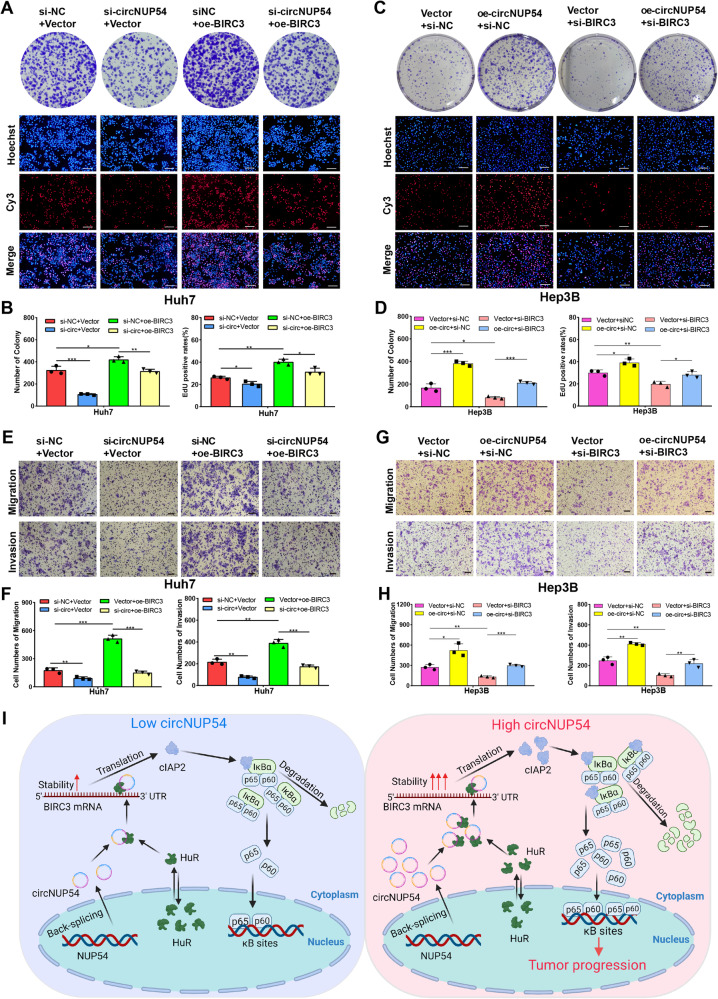
CircNUP54 promotes HCC progression by targeting BIRC3 via HuR. **A**, **B** Rescue experiments based on colony formation and EdU showed that BIRC3 overexpression reversed the proliferative suppression caused by circNUP54 knockdown in Huh7 cells. **C**, **D** Rescue experiments based on colony formation and EdU revealed that BIRC3 knockdown counteracted the proliferative facilitation induced by circNUP54 overexpression in Hep3B cells. Scale bar = 100 μm. **E**, **F** Transwell assays revealed that transfection of the BIRC3 upregulation plasmid rescued the suppressive effect of si-circNUP54 on cell migration and invasion in Huh7 cells. **G**, **H** Transwell assays revealed that transfection of si-BIRC3 restored the promotive effect of the circNUP54 upregulation plasmid on cell migration and invasion in Hep3B cells. **I** Schematic diagram illustrates the mechanism of circNUP54 in HCC progression. Scale bar = 100 μm. Data presented as means ± SD of three independent experiments. *p < 0.05, **p < 0.01, ***p < 0.001 (Student’s *t*-test).

To further clarify the role of BIRC3 in the tumor-promoting effect of circNUP54, rescue experiments were conducted. Functionally, as revealed by colony formation, EdU, and Transwell assays, circNUP54 knockdown inhibited the proliferation, migration, and invasion of Huh7 cells. This inhibition was reversed by the overexpression of BIRC3 (Fig. 7A, B, E, F). Similarly, circNUP54 upregulation promoted the growth, motility, and intrusion of Hep3B cells, whereas this promotion was blocked by the deletion of BIRC3 (Fig. 7C, D, G, H). Since HuR acts as an intermediate molecule of circNPU54 and BIRC3, rescue experiments targeting HuR were also designed and performed. We found that interference of HuR could restore the circNUP54-induced HCC progression and vice versa, as verified by colony formation, EdU, and Transwell assays (Fig. S6A–H). In short, circNUP54 plays an oncogenic role in HCC cells by targeting BIRC3 via HuR (Fig. 7I).

## Discussion

HCC is an aggressive and heterogeneous malignancy characterized by an advanced stage of diagnosis and an unsatisfactory prognosis [35, 36]. In-depth investigation of the mechanism underlying the development of HCC is crucial for the identification of new therapeutic targets and the improvement of outcomes. CircRNAs are endogenously produced non-coding RNAs that exhibit high abundance and structural stability, making them promising candidates for the oncological treatment of cancer [7]. Many circRNAs related to hepatocarcinogenesis have been identified [37–39]. For example, EIF4A3-induced circTOLLIP facilitates HCC progression via upregulating PBX3 expression by sponging miR-516a-5p [40]. Exosomal circRNA-100338 enhances the metastatic ability of HCC cells [41], and N6-Methyladenosine-Modified circCPSF6 contributes to HCC malignancy by upregulating YAP1 [42]. In this study, we identified a previously unexplored circular RNA, circNUP54, which was upregulated and clinically correlated with poor HCC prognosis. Our experiments demonstrated that circNUP54 activated the NF-κB signal pathway and promoted HCC progression via regulation of HuR-mediated BIRC3 expression, the downstream gene of circNUP54. Thus, the circNUP54/HuR/BIRC3/NF-κB axis is a novel possible therapeutic target for HCC.

To date, three main mechanisms by which circRNAs exhibit their functions have been reported, including sponging miRNAs, encoding peptides, and binding to RBPs [43]. AGO2, a unique protein of the RNA-induced silencing complex, is considered to be an indispensable part of the complex by which circRNAs sponge miRNAs [44, 45]. In this study, the RIP assays precipitated by anti-AGO2 showed that circNUP54 could not be enriched, indicating that circNUP54 may not function as a “miRNA sponge.” In general, circRNAs that encode peptides have distinctive open reading frames that span the splicing site [46]. However, circNUP54 did not meet this requirement in our study. While previously less known, the interaction between circRNAs and RBPs has gained prominence. Studies have shown that circRNAs act as protein sponges, decoys, scaffolds, and recruiters by binding to specific proteins [17]. In this study, we found that circNUP54 acted as a RBP decoy by interacting with HuR and promoting its nucleocytoplasmic redistribution.

The HuR, also known as ELAVL1, predominantly localizes to the nucleus in normal tissues [47]. However, in cancerous tissues, its distribution shifts to the cytoplasm due to cytoplasmic export activation [23]. As a nucleoplasmic shuttle protein, the HNS, which is located between the second and third RRMs, governs HuR translocation [34]. HuR is overexpressed in most malignant cells and correlated with unfavorable outcomes [48], and the cytoplasmic accumulation is believed to be an essential process for its functional role [49]. The cytoplasmic HuR exhibits pro-tumorigenic function by binding to a subset of mRNAs and promoting their stabilities [50, 51], usually binding with the U- or AU- AREs located in the 3′-UTR region of the target mRNAs [52, 53]. For instance, Qiu et al. reported that over-expressed circTHBS1 binds with HuR, promotes its translocation from the nucleus to the cytoplasm, and eventually contributes to gastric cancer progression [10]. It is unknown, however, whether HuR plays a role in the development of HCC. In this study, we found that circNUP54 acts as a decoy of HuR by binding to RRM2 to facilitate its cytoplasmic accumulation, ultimately enhancing the stability of downstream BIRC3 mRNA.

CIAP2, encoded by BIRC3, is one of the eight human inhibitors of apoptosis proteins (IAPs) that contribute to apoptosis escape of cancer cells [54, 55]. CIAP2 has become an exciting research topic because of its exceedingly elevated expression in a wide range of cancers, including gallbladder cancer [27], gastric cancer [56], renal cell carcinomas [57], and, unsurprisingly, HCC [28]. Mechanistically, cIAP2 and cIAP1 are both E3 ubiquitin ligases. Moreover, cIAP1/2 forms a complex with TNF receptor 2 or 3 (TNFR2/3) in the condition of receptor-induced activation and subsequently mediates the ubiquitin-dependent degradation of inhibitor of NF-κB (IκBα), eventually activating the NF-κB signaling pathway [58–60]. CIAP2 knockdown markedly reduces the proliferation and activity of the NF-κB pathway in HCC cells [28]. In this study, we identified an upstream regulatory mechanism for BIRC3-induced NF-κB pathway activation. For the first time, we proposed that elevated BIRC3 expression in HCC is caused by circNUP54-mediated HuR cytoplasmic export. Our study confirmed that BIRC3 is the major participant in the circNUP54-driven carcinogenesis of HCC cells. However, further investigation is needed to determine if other unknown pathways are involved in BIRC3-induced NF-κB pathway activation, as the regulation of the downstream can be multilayered and multidirectional.

In conclusion, our results demonstrate that upregulated circNUP54 contributes to HCC carcinogenesis and predicts unfavorable outcomes. Mechanistically, circNUP54 binds with HuR and facilitates its cytoplasmic accumulation, stabilizing BIRC3 mRNA and eventually activating the NF-κB signal pathway. These findings indicate that the circNUP54/HuR/BIRC3/NF-κB axis may be a promising therapeutic target for HCC.

### Supplementary information

Legends of supplementary figures

Figure S1

Figure S2

Figure S3

Figure S4

Figure S5

Figure S6

supplementary tables

original data

supplementary Excel

aj-checklist

## Data Availability

All data used to support the findings of this study are available from the corresponding author upon request.
