# Supplementary material for: CircNUP54 promotes hepatocellular carcinoma progression via facilitating HuR cytoplasmic export and stabilizing BIRC3 mRNA
Source: Cell Death Dis. 2024 Mar 5;15(3):191. doi: 10.1038/s41419-024-06570-4 (PMC10914787; doi:10.1038/s41419-024-06570-4)
Supplement: Supplementary file 1 — Legends of supplementary figures [file 41419_2024_6570_MOESM1_ESM.doc]

**Supplementary Figure 1. CircNUP54 overexpression promotes HCC cell proliferation, migration, and invasion *in vitro*. A** Schematic diagrams of the three most highly expressed circRNAs (circMED13L, circNUP54, and circATXN1). CircMED13L and circATXN1 originate from exon 2 and exons 2–4 of their host genes, respectively. Exons 2–5 of the parental gene NUP54 comprised circNUP54. **B** Schematic illustration of circNUP54 siRNAs (si-circNUP54#1 and si-circNUP54#2) targeting the back-splicing site of circNUP54. **C** qRT-PCR analysis of circNUP54 and NUP54 mRNA in Hep3B cells transfected with the indicated lentiviruses. **D** The CCK-8 assay was used to assess the proliferation of Hep3B cells transfected with the vector or circNUP54. **E**, **F** Colony formation assays verified the cell colony formation ability. **G**, **H** The cell growth-promoting effect of circNUP54 was determined using an EdU assay. Scale bar = 100 μm. **I**, **J** Wound-healing experiments were performed to detect the migratory ability of transfected Hep3B cells. Scale bar = 200 μm. **K**–**L** Transwell assays in Hep3B cells overexpressing circNUP54 to evaluate migration and invasion abilities. Scale bar = 100 μm. Data presented as means ± SD of three independent experiments. **p <* 0.05, ***p <* 0.01 (Student’s *t*-test).

**Supplementary Figure 2. CircNUP54 promotes HCC metastasis *in vivo***. **A**, **B** Pictures of subcutaneous tumors in nude mice after injection of Huh7 (Lv-sh-NC, Lv-sh-circNUP54) or Hep3B cells (Lv-vector, Lv-circNUP54) (n = 6). **C, D** qRT-PCR analysis of circNUP54 level in the subcutaneous tumors. **E** HE staining images of orthotopic xenograft tumors from the indicated groups. Tumors with circNUP54-overexpression possessed a more tortuous margin. Left scale bar = 200 μm, right scale bar = 25 μm. **F** The picture of the *ex vivo* lungs of sacrificed mice that received tail vein injections of the indicated Huh7 cells (Lv-sh-NC, Lv-sh-circNUP54, or Lv-circNUP54). **G** Statistical number graph of lung metastatic lesions from the *ex vivo* lungs. **H** HE staining of lung metastatic nodules. Scale bar = 1000 μm, up; scale bar = 100 μm, down. Data presented as means ± SD of three independent experiments. **p <* 0.05, ****p <* 0.001 (Student’s *t*-test).

**Supplementary Figure 3. A**, **B** KEGG and GO enrichment of the up-regulated genes after circNUP54 knockdown. **C** The expression of AKT3, LTB, and CXCL3 in HCC and normal liver tissue (TCGA database). **D**, **E** qRT-PCR verifying the efficiency of siRNAs (si-NC and si-BIRC3) or plasmids (Vector or BIRC3) in transfected cells. **F** WB showing cIAP2 expression in SNU449 cells after transfection with siRNAs (si-NC and si-BIRC3) or plasmids (Vector or BIRC3). Data presented as means ± SD of three independent experiments. **p <* 0.05, ***p <* 0.01, ****p <* 0.001 (Student’s *t*-test).

**Supplementary Figure 4.** **A** The predicted binding regions of circNUP54 and HuR according to CatRAPID. **B** The amino acid sequence of HuR RNA recognition motifs (RRMs). The most likely sites of HuR binding to circNUP54 are sites 126–177, which are located at RRM2. This is consistent with our findings.

**Supplementary Figure 5.** **A**, **B** qRT-PCR analysis of HuR mRNA in Huh7 and SNU449 cells after transfection with siRNAs (si-NC and si-HuR) or plasmids (Vector or oe-HuR). **C**, **D** Quantitative WB analysis of cIAP2 after HuR knockdown or overexpression in Huh7 and SNU449 cells. **E** The HuR binding sites (the bottom purple vertical lines) in the BIRC3 3’UTR region predicted by RBPmap. **F** Correlation analysis of HuR and BIRC3 expression according to TCGA. **G** BIRC3 3’UTR probe pulldown was performed in SNU449 cells with circNUP54 overexpression or knockdown. The pulled-down HuR was detected using WB. **H**, **I** Quantitative WB analysis of cIAP2 in cells with indicated plasmid transfection. **J** The motif of HuR. **K**, **L** Outlook of subcutaneous xenografts at the endpoint. **M** Growth curves of subcutaneous tumors in nude mice (n = 5). **N** Weight of tumors dissected from the mice. Data presented as means ± SD of three independent experiments. ***p <* 0.01, ****p <* 0.001, *****p <* 0.0001 (Student’s *t*-test).

**Supplementary Figure 6. CircNUP54 promotes HCC progression via HuR. A**, **B** Colony formation and EdU assays demonstrated that HuR overexpression reversed the proliferative suppression caused by circNUP54 knockdown in Huh7 cells. Scale bar = 100 μm. **C**, **D** HuR knockdown counteracted the proliferative promotion induced by circNUP54 overexpression in Hep3B cells. **E**, **F** Transwell assay showed that HuR upregulation rescued the suppressive effect of si-circNUP54 on cell migration and invasion in Huh7 cells. **G**, **H** Transfection of si-HuR restored the promotive effect of the circNUP54 upregulation plasmid on cell migration and invasion in Hep3B cells. Scale bar = 100 μm. Data presented as means ± SD of three independent experiments. **p* < 0.05, ***p* < 0.01, *** *p*< 0.001 (Student’s *t*-test).
