# Supplementary figures and images for: CircNUP54 promotes hepatocellular carcinoma progression via facilitating HuR cytoplasmic export and stabilizing BIRC3 mRNA
Source: Cell Death Dis. 2024 Mar 5;15(3):191. doi: 10.1038/s41419-024-06570-4 (PMC10914787; doi:10.1038/s41419-024-06570-4)

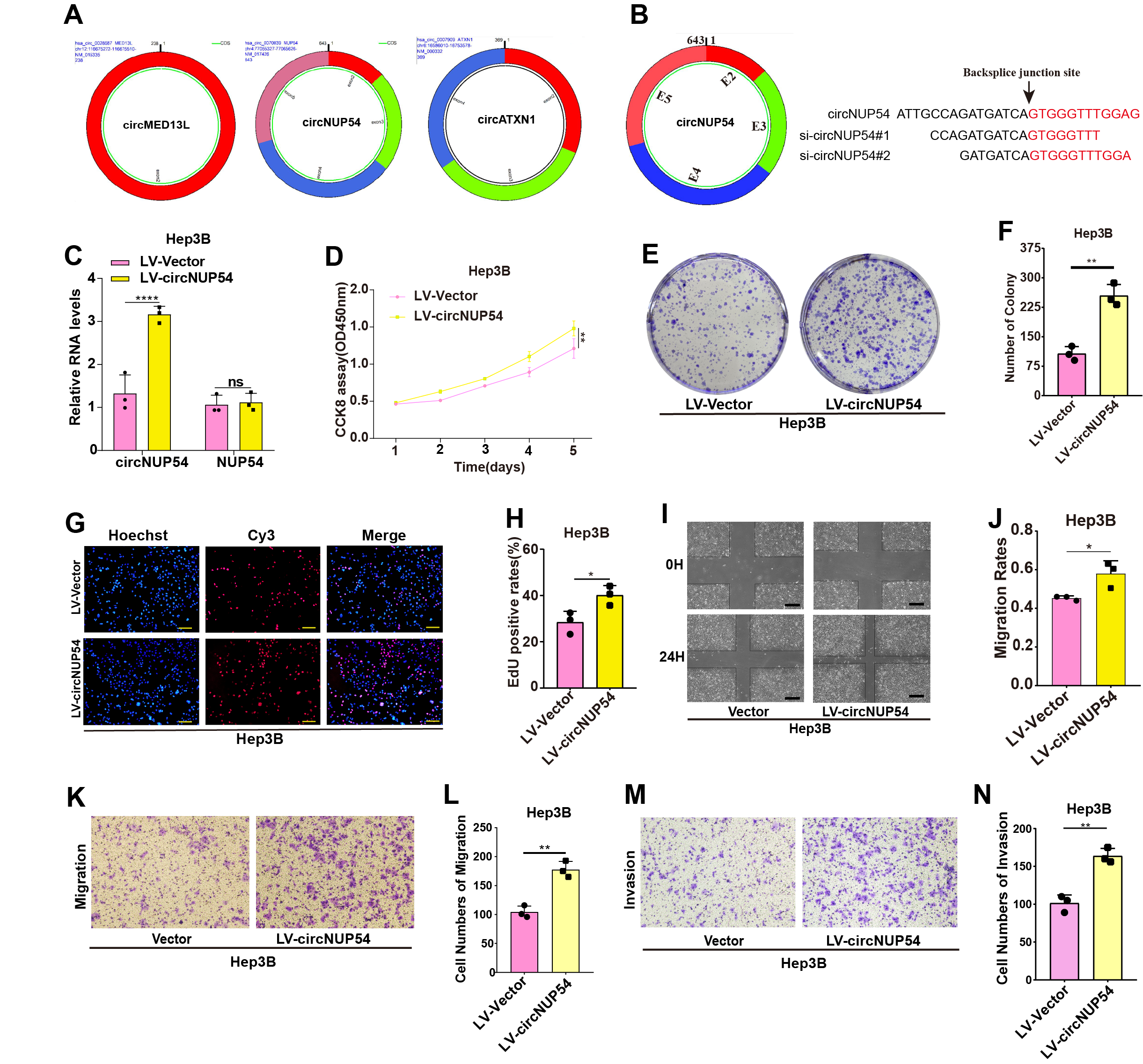

Supplement: Supplementary file 2 — Figure S1 [file 41419_2024_6570_MOESM2_ESM.png]

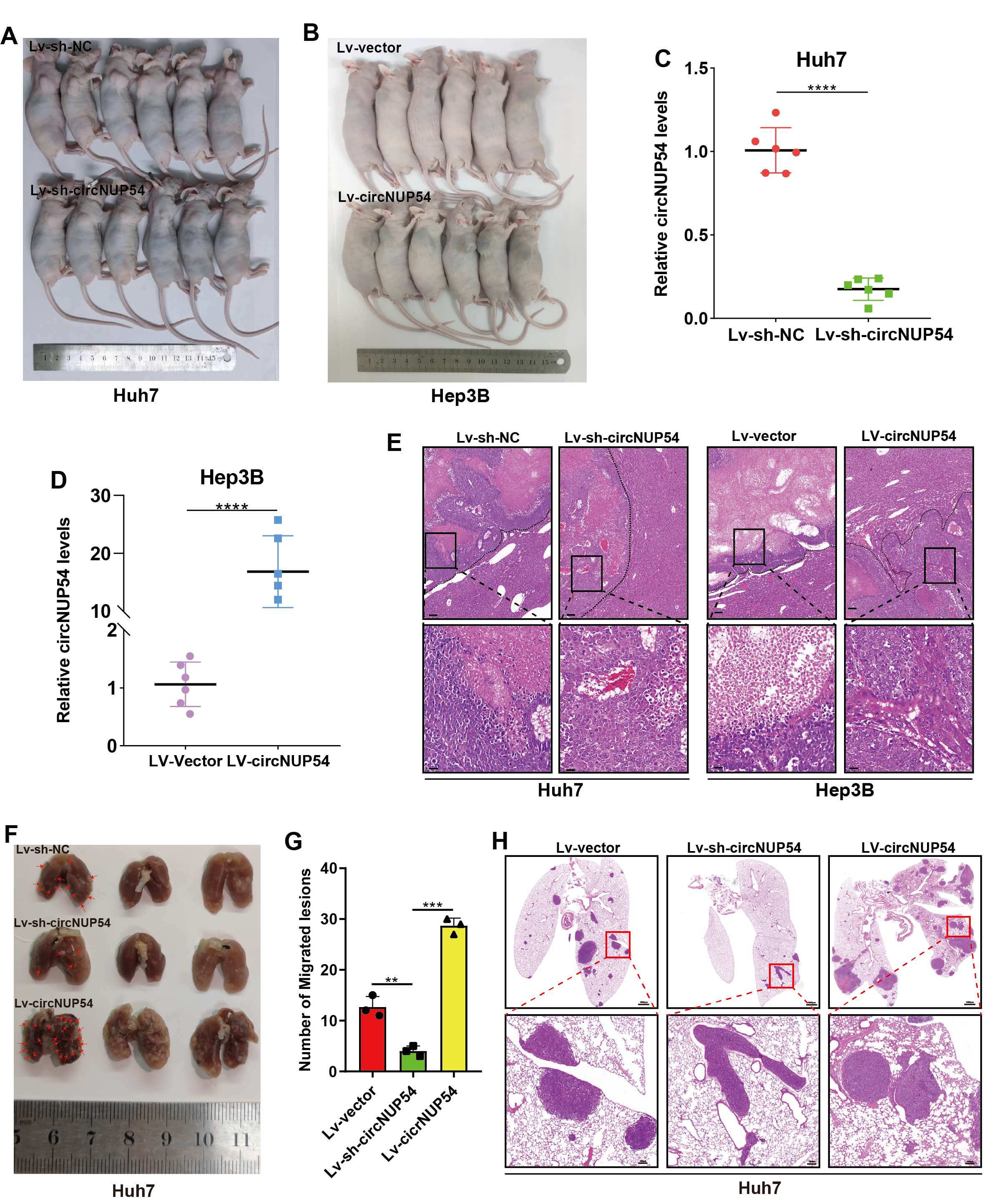

Supplement: Supplementary file 3 — Figure S2 [file 41419_2024_6570_MOESM3_ESM.png]

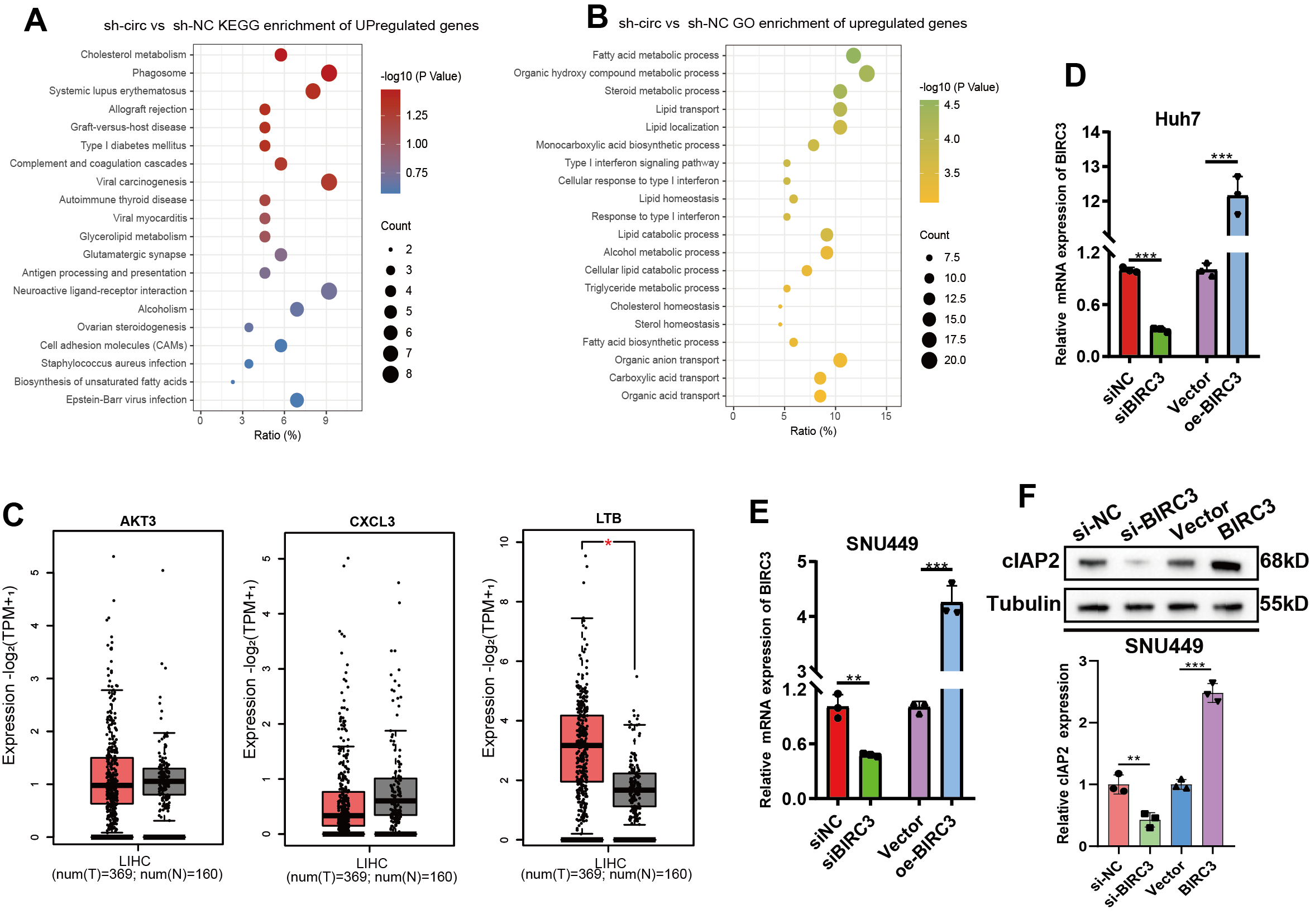

Supplement: Supplementary file 4 — Figure S3 [file 41419_2024_6570_MOESM4_ESM.png]

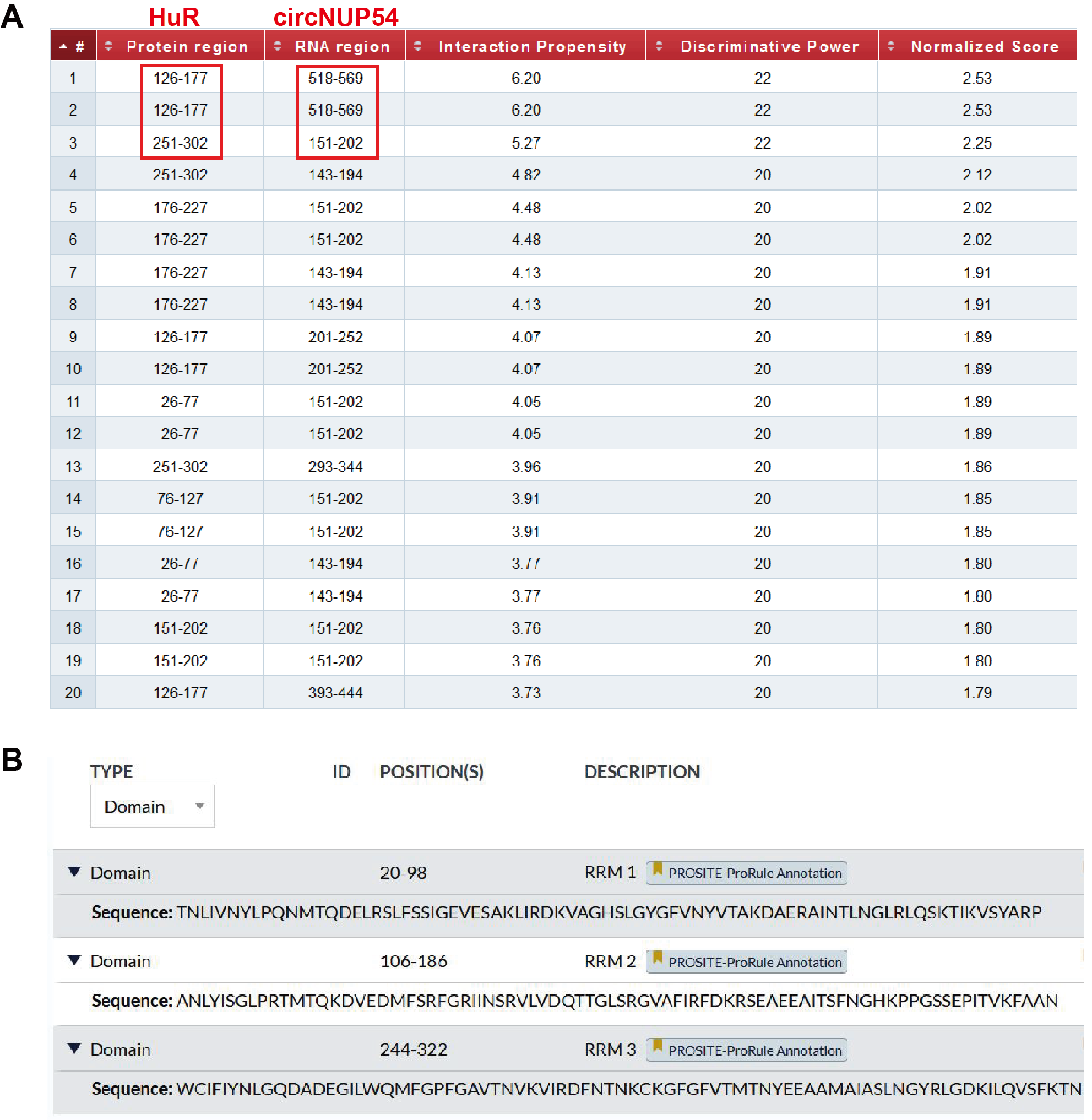

Supplement: Supplementary file 5 — Figure S4 [file 41419_2024_6570_MOESM5_ESM.png]

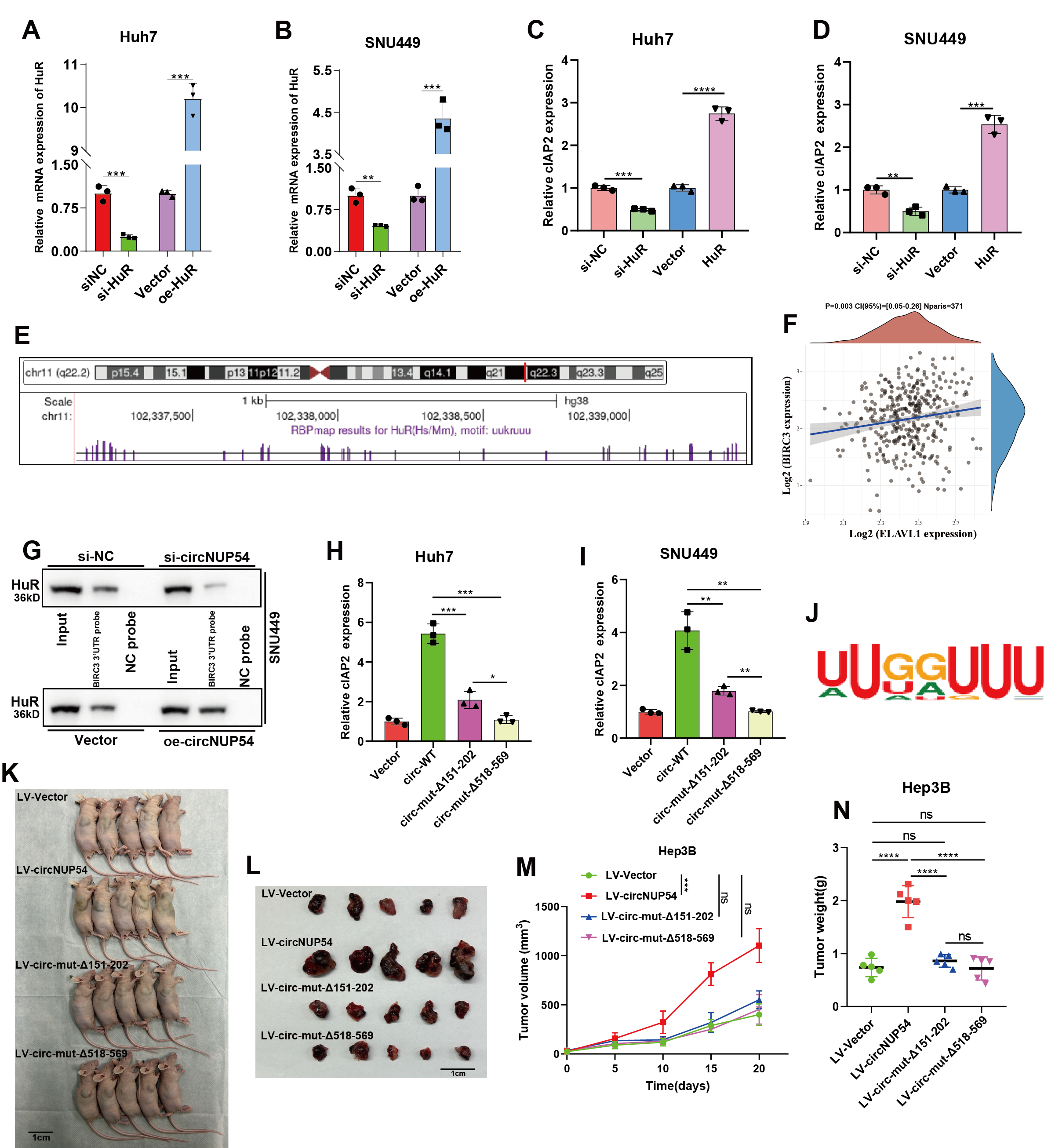

Supplement: Supplementary file 6 — Figure S5 [file 41419_2024_6570_MOESM6_ESM.png]

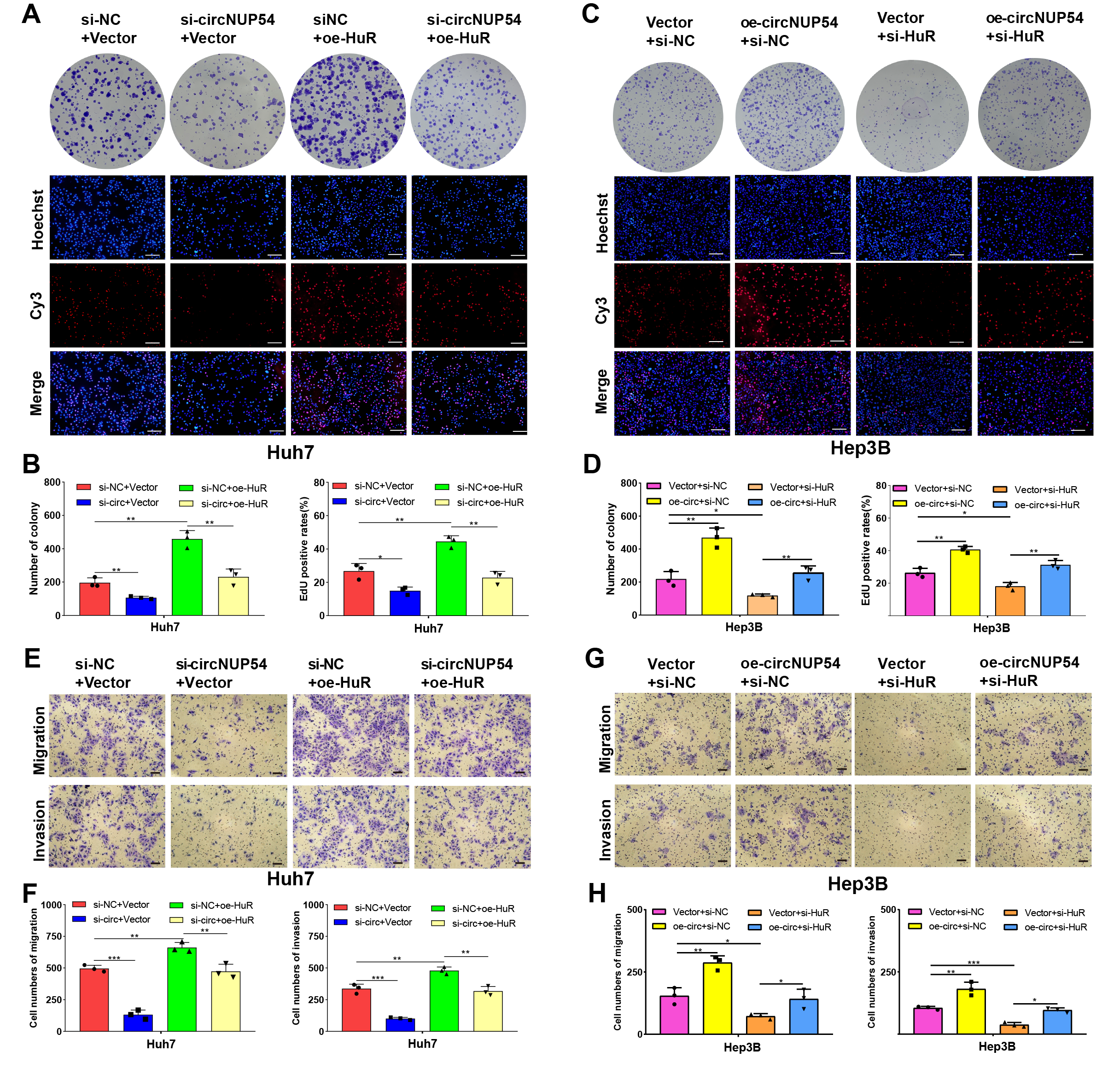

Supplement: Supplementary file 7 — Figure S6 [file 41419_2024_6570_MOESM7_ESM.png]
