# Supplementary material for: CircNUP54 promotes hepatocellular carcinoma progression via facilitating HuR cytoplasmic export and stabilizing BIRC3 mRNA
Source: Cell Death Dis. 2024 Mar 5;15(3):191. doi: 10.1038/s41419-024-06570-4 (PMC10914787; doi:10.1038/s41419-024-06570-4)
Supplement: Supplementary file 8 — supplementary tables [file 41419_2024_6570_MOESM8_ESM.docx]

**Supplementary Table 1. Clinicopathological information of 68 patients with HCC.**

| **Clinicopathological Characteristics** | **Number of cases (%)** | **Clinicopathological Characteristics** | **Number of cases (%)** |
| --- | --- | --- | --- |
| Age (years) |  | Gender |  |
| ≥ 60 | 23 (33.8%) | Male | 59 (86.8%) |
| < 60 | 45 (66.2%) | Female | 9 (13.2%) |
| Cirrhosis |  | AFP (μg/L） |  |
| Yes | 30 (44.1%) | ≥ 400 | 38 (55.9%) |
| No | 38 (55.9%) | < 400 | 30 (44.1%) |
| HBV infection |  | Tumor size |  |
| Yes | 55 (80.9%) | >5 cm | 45 (66.2%) |
| No | 13 (19.1%) | ≤5 cm | 23 (33.8%) |
| Histological grade |  | TNM stage |  |
| G1 | 5 (7.4%) | Ⅰ | 10 (14.7%) |
| G2 | 26 (38.2%) | Ⅱ | 11(16.2%) |
| G3 | 37 (54.4%) | Ⅲ | 43 (32.2%) |
| BCLC stage |  | Ⅳ | 4 (5.9%) |
| A | 10 (14.7%) | MVI |  |
| B | 10 (14.7%) | Yes | 44 (64.7%) |
| C | 48 (70.6%) | No | 24 (35.3%) |

MVI: microvascular invasion; BCLC: Barcelona Clinic Liver Cancer; AFP: alpha-fetoprotein

**Supplementary Table 2. The sequence of primer, probe, and siRNA used in this study**

| **Gene** | **Sequence (5’-3’)** | **Application** |
| --- | --- | --- |
| circNUP54-F | GGAGGAAACCAGACCCTTACT | qRT-PCR |
| circNUP54-R | GAGTCCAGTAGTGCCTGTGT |  |
| NUP54-F | TTGGCACAACAACGGGAACTA |  |
| NUP54-R | CAGAAAGAGCACTCGCAGTATT |  |
| circMED13L-F | CTGCACGGATGGTCTTGAAA |  |
| circMED13L-R | CTGGTGACTTGATGCACGATG |  |
| circATXN1-F | CTGCTTTGTGTATGGCGTCG |  |
| circATXN1-R | TGCAGACAGCGGATGAAACT |  |
| BIRC3-F | TTTCCGTGGCTCTTATTCAAACT |  |
| BIRC3-R | GCACAGTGGTAGGAACTTCTCAT |  |
| HuR/ELAVL1-F | AACTACGTGACCGCGAAGG |  |
| HuR/ELAVL1-R | CGCCCAAACCGAGAGAACA |  |
| LTB-F | GTACGGGCCTCTCTGGTACA |  |
| LTB-R | GTCCACCATATCGGGGTGAC |  |
| AKT3-F | AATGGACAGAAGCTATCCAGGC |  |
| AKT3-R | TGATGGGTTGTAGAGGCATCC |  |
| CXCL3-F | CCAAACCGAAGTCATAGCCAC |  |
| CXCL3-R | TGCTCCCCTTGTTCAGTATCT |  |
| GAPDH-F | AGAAGGCTGGGGCTCATTTG |  |
| GAPDH-R | AGGGGCCATCCACAGTCTTC |  |
| U6-F | CTCGCTTCGGCAGCACA |  |
| U6-R | AACGCTTCACGAATTTGCGT |  |
| si-circNUP54#1 | CCAGATGATCAGTGGGTTT | siRNA |
| si-circNUP54#2 | GATGATCAGTGGGTTTGGA |  |
| si-circNUP54#3 | GATCAGTGGGTTTGGAGGA |  |
| si-circNUP54-NC | ACGTGCCACATGGTCGACGT |  |
| si-HuR | GACCCAGGATGAGTTACGA |  |
| si-HuR-NC | CGGACGGCGAAATCCGCCT |  |
| si-BIRC3 | CCTGGATAGTCTACTAACT |  |
| si-BIRC3-NC | ATCCAGGGCACCGGCCTATG |  |
| circNUP54 probe | CATTGCCAGATGATCAGTGGGTTTGGAGGAT -/3bio/ | RNA pull down |
| circNUP54 NC probe | ATCCTCCAAACCCACTGATCATCTGGCAATG -/3bio/ |  |
| BIRC3 3’ UTR probe | CTAATTTGGTTTCCTTAAAATTTTTATTTATTTACAACTCAAAAAACATTGTTTT- /3bio/ |  |
| BIRC3 3’ UTR NC probe | ATCCTTTCTAGGCCAATTCCCGGGATCGATC -/3bio/ |  |
| CircNUP54 probe | /5CY3/- ATCCTCCAAACCCACTGATCATCTGGCAATG | FISH |
| 18S probe | /5CY3/-CTTCCTTGGATGTGGTAGCCGTTTC |  |
| NC probe | /5CY3/-TGCTTTGCACGGTAACGCCTGTTTT |  |

**Supplementary Table 3. Antibodies used in this study.**

| **product** | **Source** | **No. of catalogue** | **Application** |
| --- | --- | --- | --- |
| anti-GAPDH | Cell Signaling Technology | 97166S | Western blot  Primary antibody |
| Anti-α-Tubulin | Cell Signaling Technology | 3873S |  |
| Anti-cIAP2 | ABclonal Technology | A0833 |  |
| Anti-p-p65-S536 | ABclonal Technology | AP1294 |  |
| Anti-t-p65 | ABclonal Technology | A22331 |  |
| Anti-p-IκBα-S32 | ABclonal Technology | AP0707 |  |
| Anti-T-IκBα | ABclonal Technology | A19714 |  |
| anti-Histone 2B | ABclonal Technology | A19812 |  |
| HRP-Goat Anti-Mouse | Epizyme Biomedical Technology | LF101 | Western blot  secondary antibody |
| HRP-Goat Anti-Rabbit | Epizyme Biomedical Technology | SA00001-1 |  |
| Anti-HuR | ABclonal Technology | A19622 | WB, IF, RIP |
| Anti-Argonaute-2 | ABclonal Technology | A19709 | RIP |
| Anti-Ki67 | Abcam | ab15580 | IHC |
| 488-conjugated Goat anti-Rabbit | Proteintech | SA00013-2 | IF secondary antibody |
| 594-conjugated Goat anti-Mouse | Proteintech | SA00013-3 |  |

**Supplementary table 4. Correlation between circNUP54 expression and clinicopathological features in HCC tissues (n = 68, χ2-test).**

| **Characteristics** | | **Low circNUP54**  **(n = 34)** | **High circNUP54**  **(n = 34)** | **P value** |
| --- | --- | --- | --- | --- |
| Age (year) | < 60 | 23 | 22 | 0.798 |
|  | ≥ 60 | 11 | 12 |  |
| Gender | Male | 29 | 30 | 0.720 |
|  | Female | 5 | 4 |  |
| AFP | < 400 | 21 | 9 | **0.003** |
|  | ≥ 400 | 13 | 25 |  |
| HBV infection | Yes | 30 | 25 | 0.123 |
|  | No | 4 | 9 |  |
| BCLC stage | A-B | 14 | 5 | **0.015** |
|  | C | 20 | 29 |  |
| TNM stage | I-II | 15 | 5 | **0.008** |
|  | III-IV | 19 | 29 |  |
| Histological grade | G1-G2 | 21 | 10 | **0.007** |
|  | G3 | 13 | 24 |  |
| Tumor size | >5 cm | 17 | 28 | **0.005** |
|  | ≤5 cm | 17 | 6 |  |
| MVI | Yes | 21 | 23 | 0.621 |
|  | No | 13 | 11 |  |
| Cirrhosis | Yes | 14 | 16 | 0.625 |
|  | No | 20 | 18 |  |

MVI: microvascular invasion; BCLC: Barcelona Clinic Liver Cancer; AFP: alpha-fetoprotein

**Supplementary table 5. Univariate and Multivariate analysis of various prognostic characteristics for overall survival (OS) in patients with HCC.**

| **Factor** | **Group** | **Univariate analysis (OS)** | | | **Multivariate analysis (OS)** | | |
| --- | --- | --- | --- | --- | --- | --- | --- |
|  |  | **P** | **HR** | **95% CI for HR** | **P** | **HR** | **95% CI for HR** |
| Age | ≥ 60/< 60 | 0.531 | 1.216 | 0.658-2.246 |  |  |  |
| Gender | Male/Female | 0.651 | 1.241 | 0.488-3.155 |  |  |  |
| Tumor size | >5cm/≤5 cm | **0.002** | 3.307 | 1.566-6.986 | 0.247 | 1.669 | 0.701-3.974 |
| HBV infection | Yes/no | 0.915 | 1.043 | 0.483-2.205 |  |  |  |
| Histological grade | G3/G1-G2 | 0.433 | 1.275 | 0.695-2.339 |  |  |  |
| TNM stage | III-IV/I-II | **<0.001** | 4.825 | 0.090-11.139 | **0.046** | 3.094 | 1.023-9.362 |
| BCLC stage | C/A-B | **0.001** | 2.915 | 1.290-6.587 | 0.986 | 1.009 | 0.361-2.823 |
| AFP | ≥ 400/< 400 | **0.015** | 2.255 | 1.174-4.330 | 0.402 | 1.357 | 0.664-2.770 |
| Cirrhosis | Yes/No | 0.621 | 0.858 | 0.468-1.574 |  |  |  |
| CircNUP54 | High/low | **0.019** | 2.188 | 1.139-4.204 | 0.884 | 1.508 | 0.498-2.249 |
| MVI | Yes/No | 0.077 | 1.787 | 0.940-3.397 |  |  |  |
| Star lesion | Yes/No | 0.174 | 1.583 | 0.816-3.069 |  |  |  |

HR: Hazard Ratio, CI: Confidence Interval, MVI: microvascular invasion; BCLC: Barcelona Clinic Liver Cancer; AFP: alpha-fetoprotein

**Supplementary table 6. Univariate and Multivariate analysis of various prognostic characteristics for RFS (recurrence-free survival) in patients with HCC.**

| **Factor** | **Group** | **Univariate analysis (RFS)** | | | **Multivariate analysis (RFS)** | | |
| --- | --- | --- | --- | --- | --- | --- | --- |
|  |  | **P** | **HR** | **95% CI for HR** | **P** | **HR** | **95% CI for HR** |
| Age | ≥ 60/< 60 | 0.491 | 1.244 | 0.668-2.315 |  |  |  |
| Gender | Male/Female | 0.864 | 1.079 | 0.453-2.571 |  |  |  |
| Tumor size | >5cm/≤5 cm | **0.029** | 2.146 | 1.802-4.257 | 0.297 | 0.578 | 0.207-1.619 |
| HBV infection | Yes/no | 0.861 | 0.930 | 0.411-2.102 |  |  |  |
| Histological grade | G3/G1-G2 | 0.165 | 1.539 | 0.838-2.826 |  |  |  |
| TNM stage | III-IV/I-II | **0.047** | 2.052 | 1.010-4.171 | 0.612 | 1.360 | 0.415-4.545 |
| BCLC stage | C/A-B | **0.039** | 2.185 | 1.041-4.586 | 0.806 | 1.175 | 0.325-4.243 |
| AFP | ≥ 400/< 400 | **0.013** | 2.244 | 1.182-4.260 | 0.746 | 0.867 | 0.366-2.057 |
| Cirrhosis | Yes/No | 0.442 | 1.263 | 0.696-2.291 |  |  |  |
| CircNUP54 | High/low | **0.018** | 2.107 | 1.138-3.902 | 0.313 | 0.675 | 0.315-1.448 |
| MVI | Yes/No | **0.002** | 2.866 | 1.462-5.617 | **0.004** | 3.730 | 1.514-9.191 |
| Star lesion | Yes/No | **0.008** | 2.623 | 1.290-5.332 | 0.381 | 0.684 | 0.293-1.600 |

HR: Hazard Ratio, CI: Confidence Interval, MVI: microvascular invasion; BCLC: Barcelona Clinic Liver Cancer; AFP: alpha-fetoprotein
