# Supplementary material for: CircNUP54 promotes hepatocellular carcinoma progression via facilitating HuR cytoplasmic export and stabilizing BIRC3 mRNA
Source: Cell Death Dis. 2024 Mar 5;15(3):191. doi: 10.1038/s41419-024-06570-4 (PMC10914787; doi:10.1038/s41419-024-06570-4)

Fig 4K

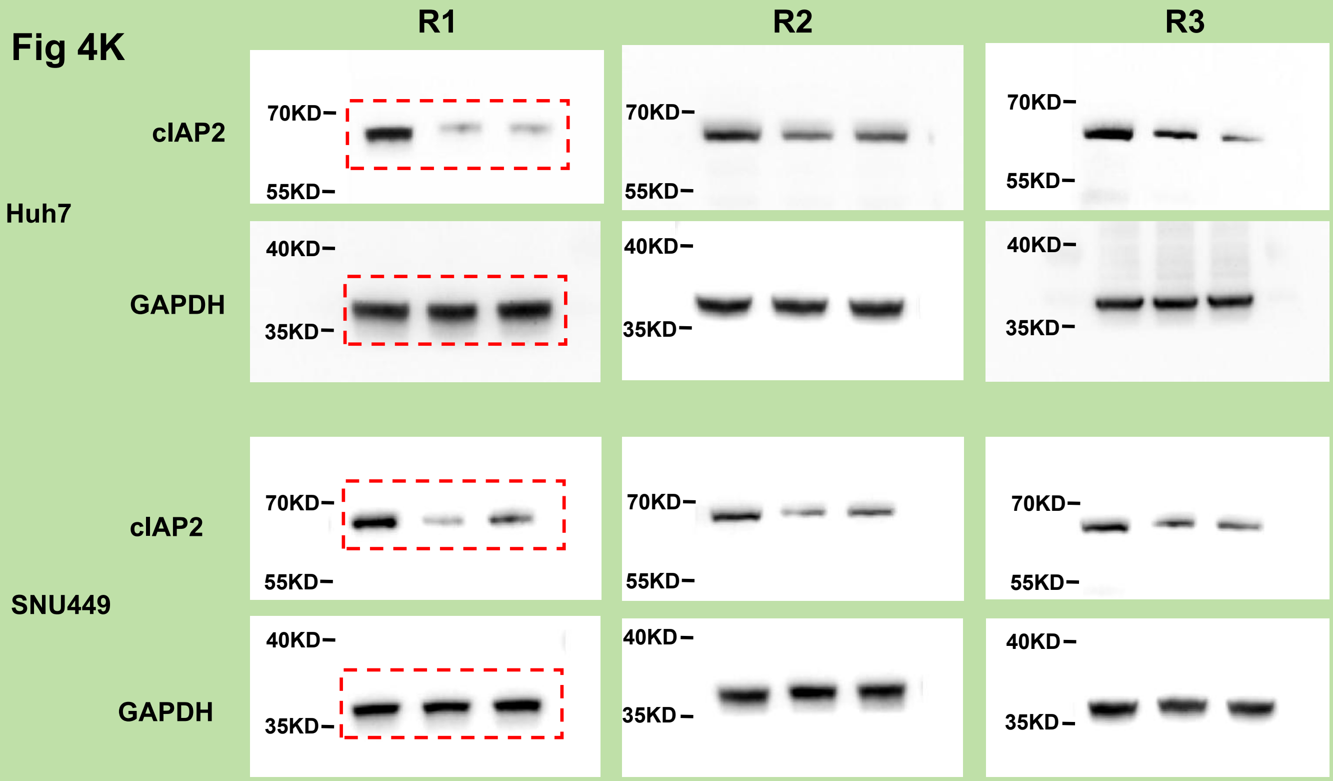
R1
R2
R3
Huh7
cIAP2
GAPDH
SNU449
cIAP2
GAPDH
70KD
55KD
40KD
35KD

Fig 4L

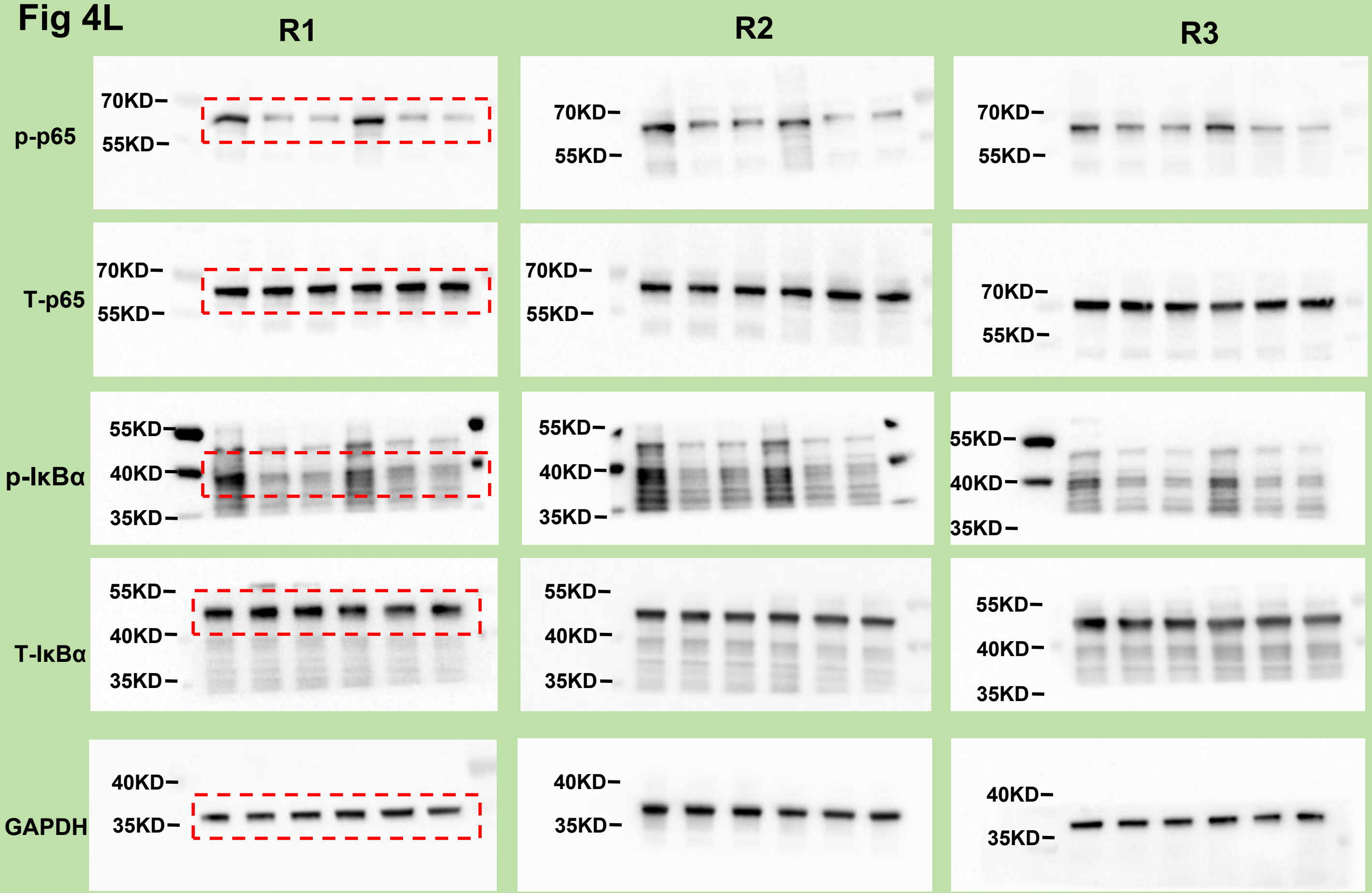
R1
R2
R3
p-p65
T-p65
p-IκBα
T-IκBα
GAPDH
70KD
55KD
40KD
35KD

Fig 4M

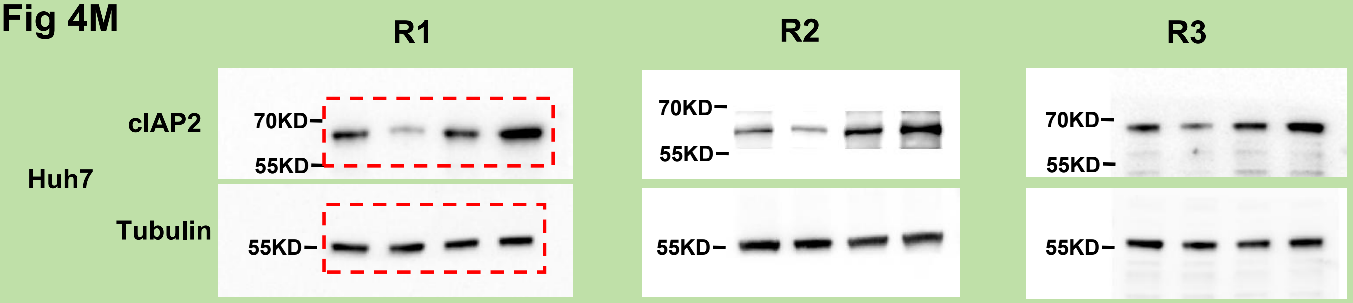

Fig S3F

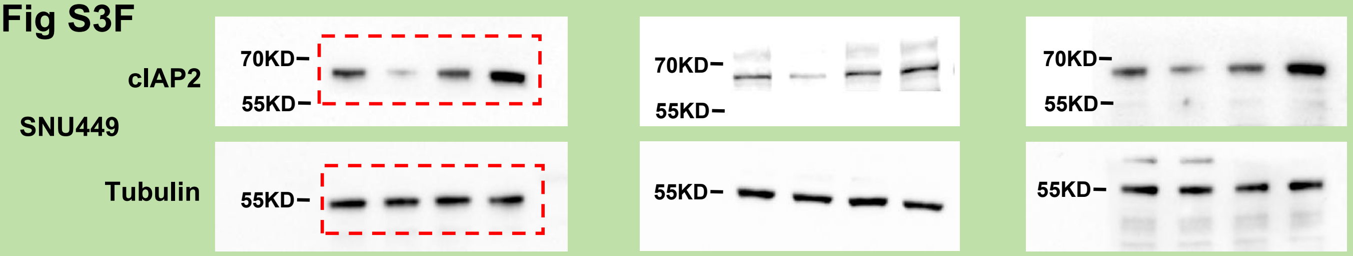

Fig 4N

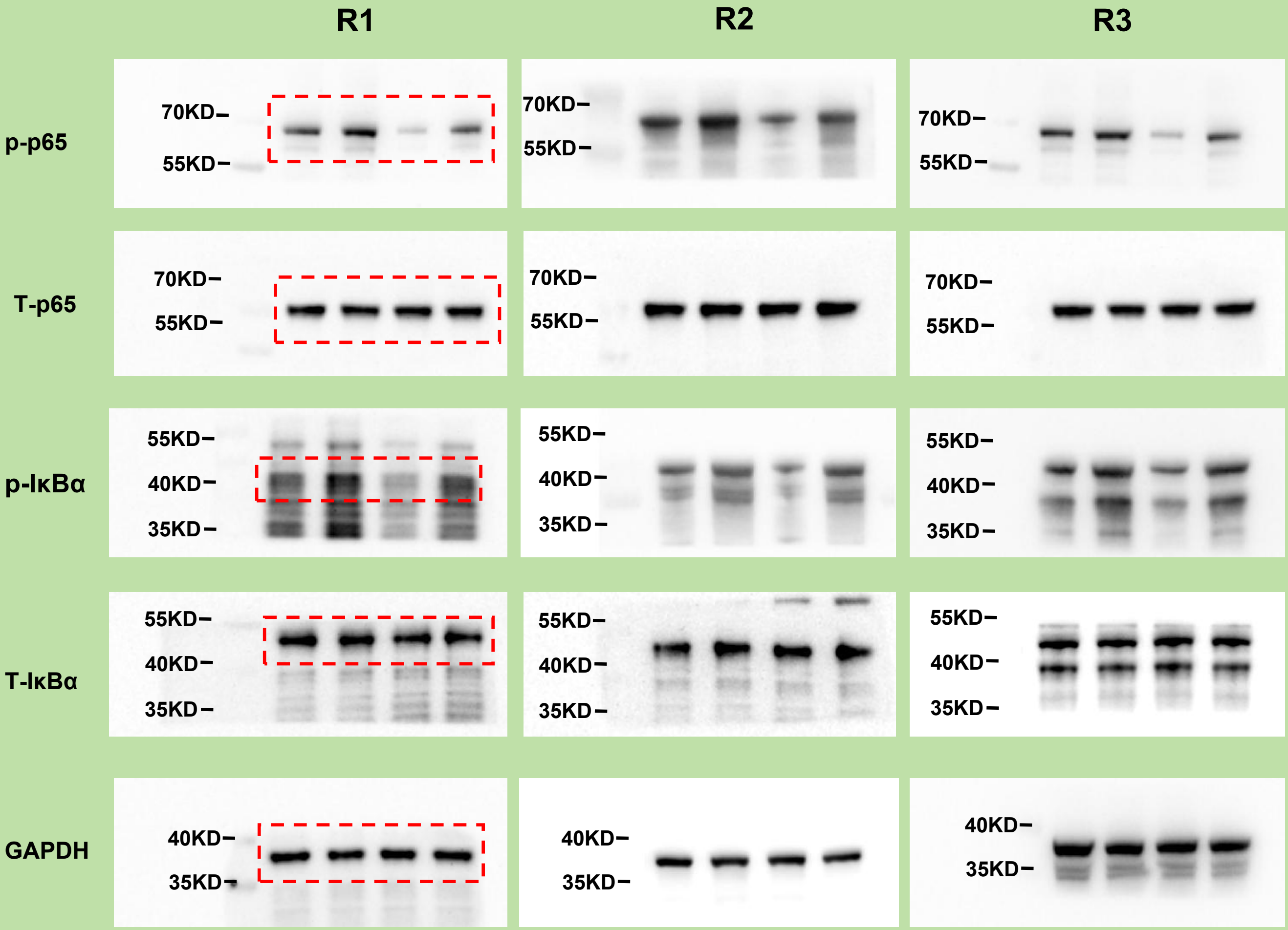

Fig 4O

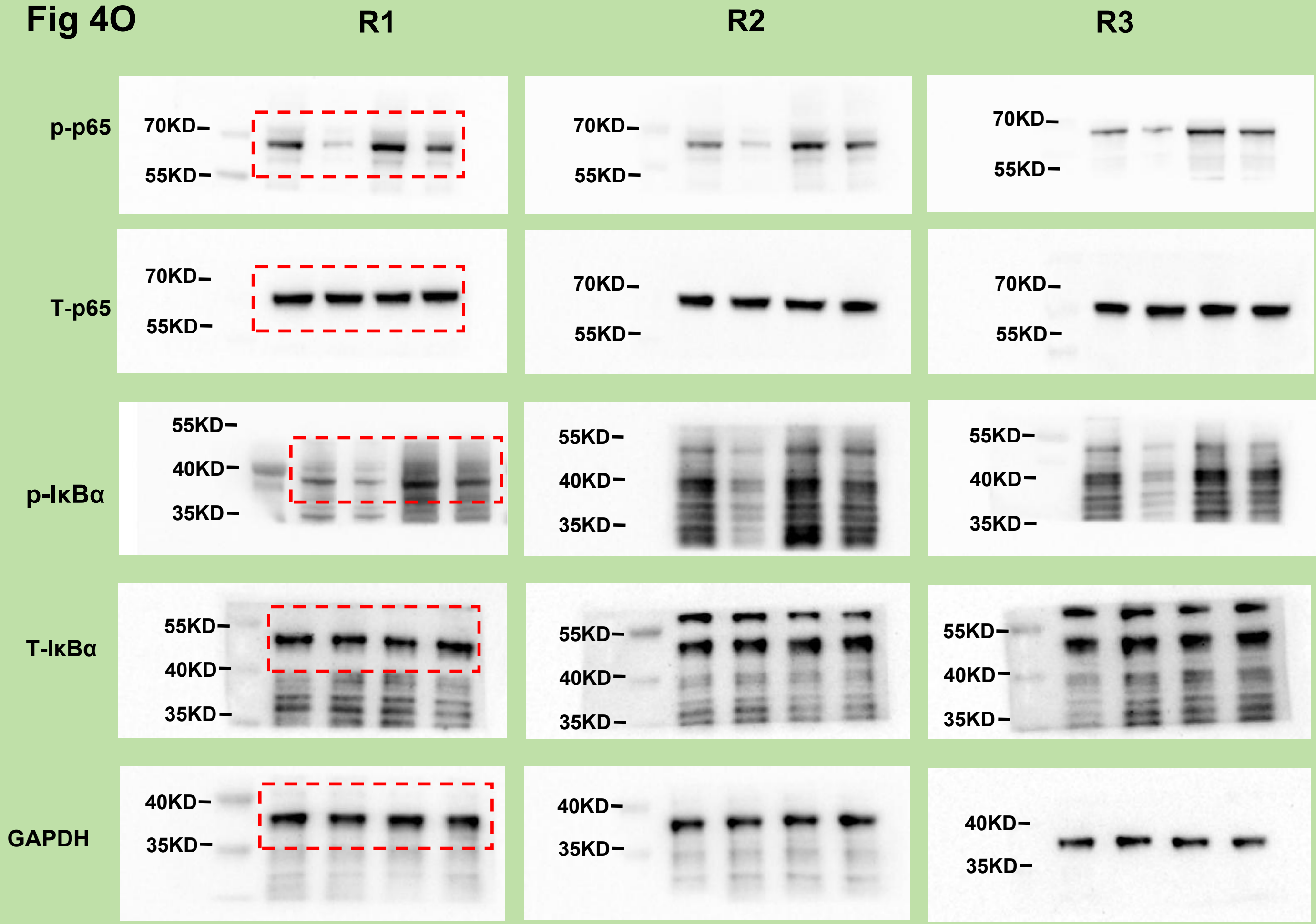

Fig 5E

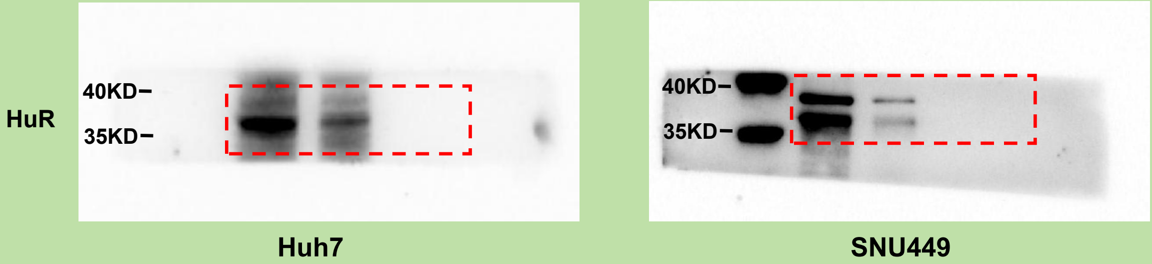

Fig 5G

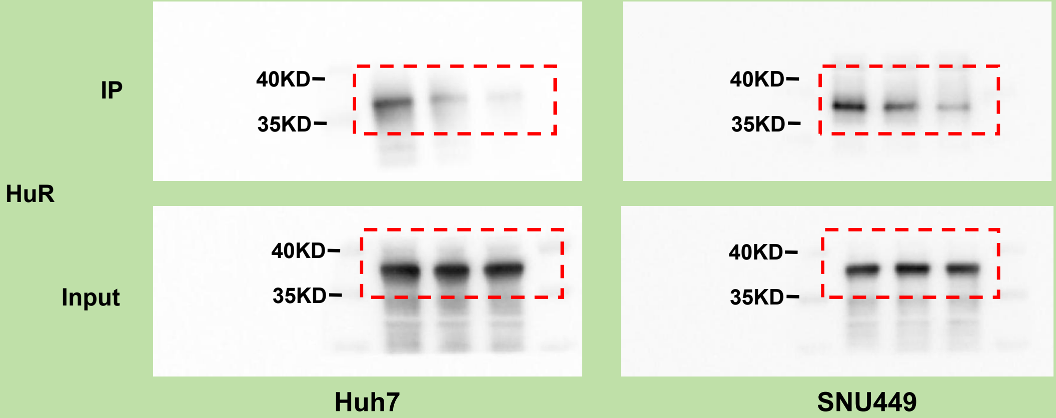

Fig 5I

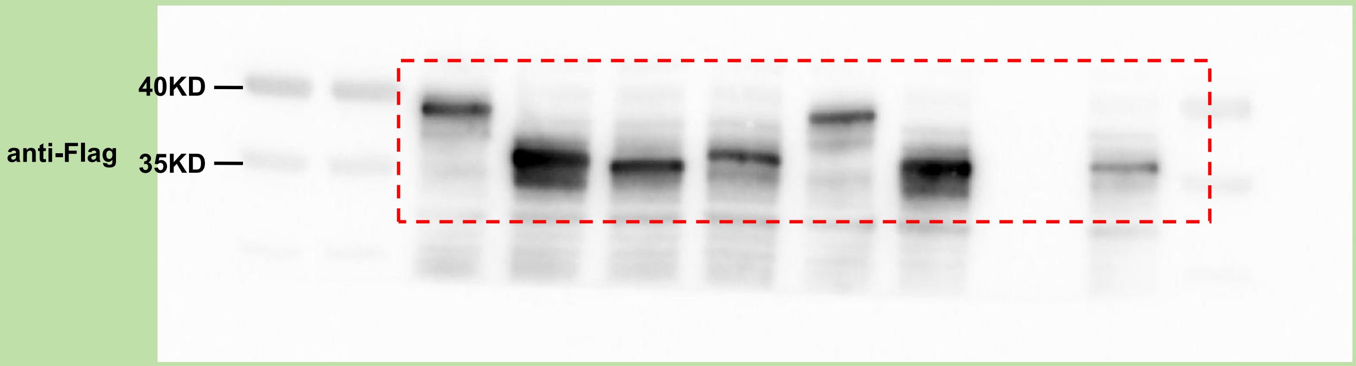
anti-Flag
40KD
35KD

Fig 5K

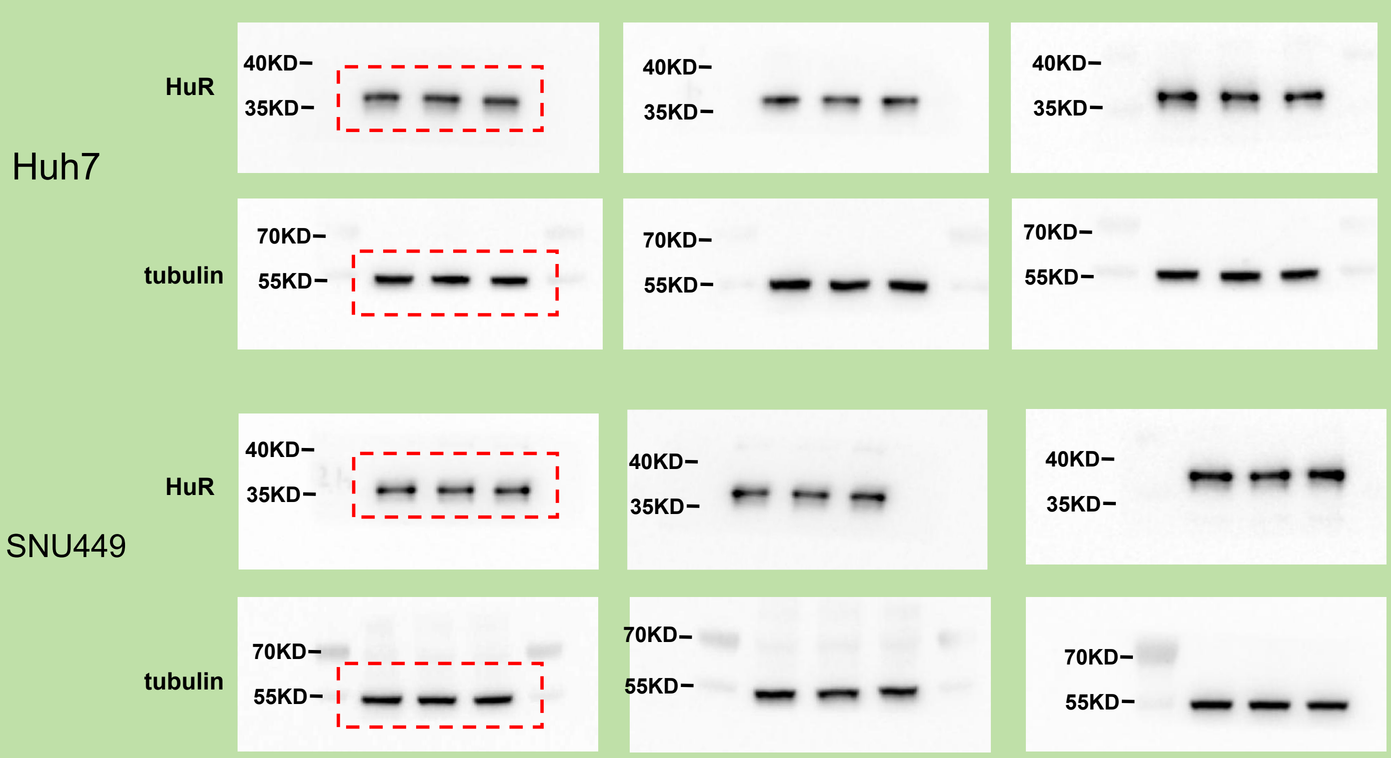
R1
R2
R3
Huh7
HuR
tubulin
SNU449
HuR
tubulin
40KD
35KD
70KD
55KD

Fig 6B

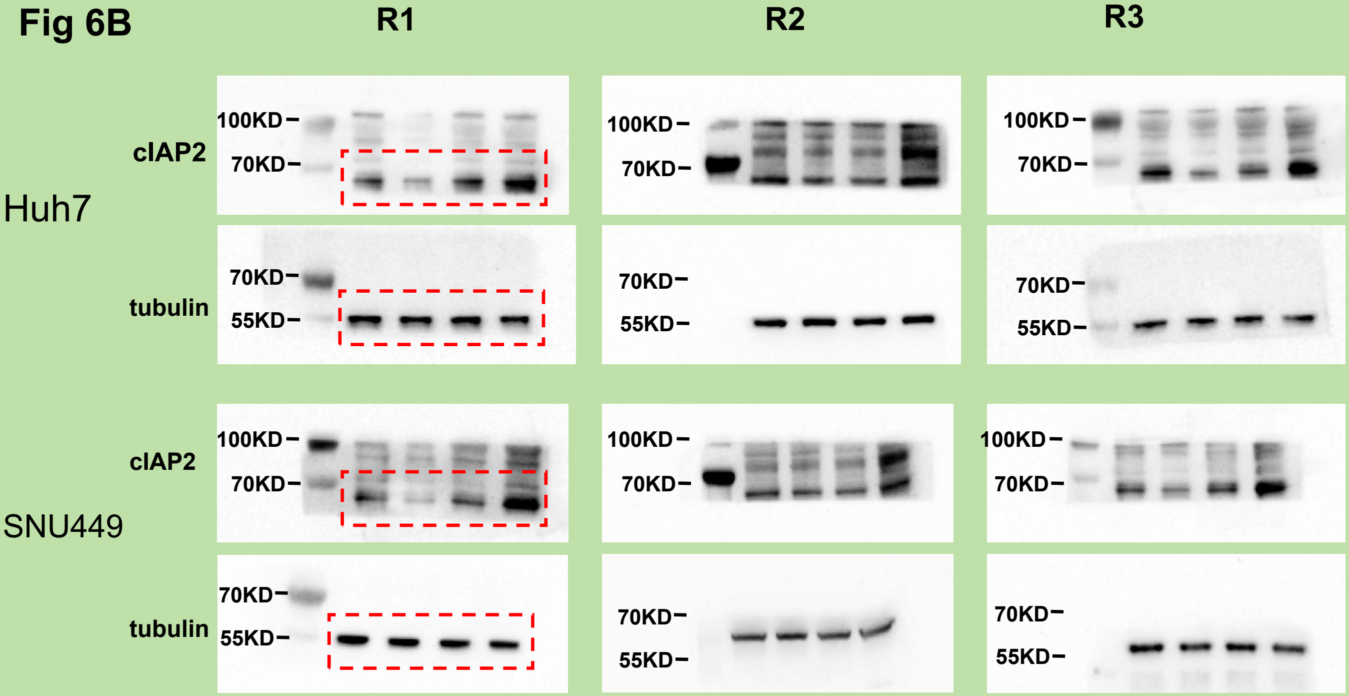

Fig 6D

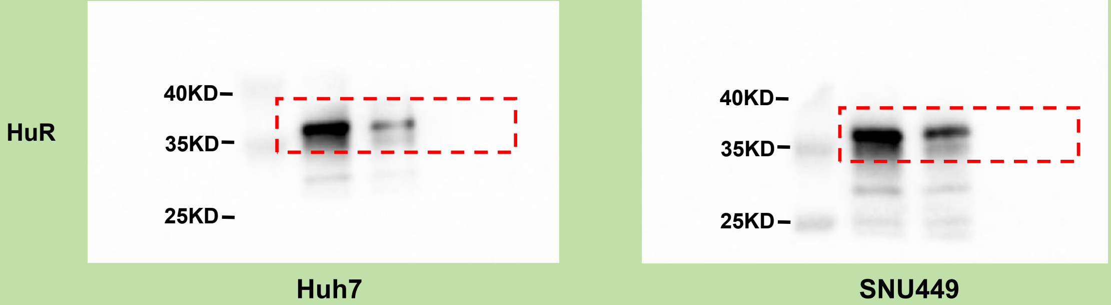

Fig 6F

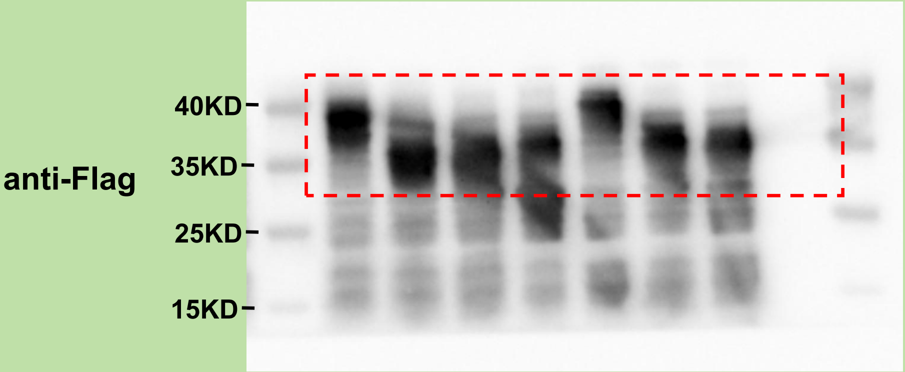

Fig 6L

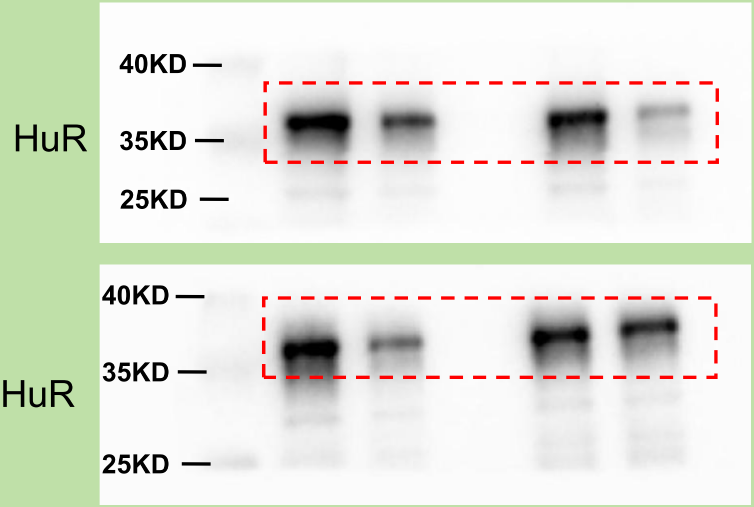

Fig S5E

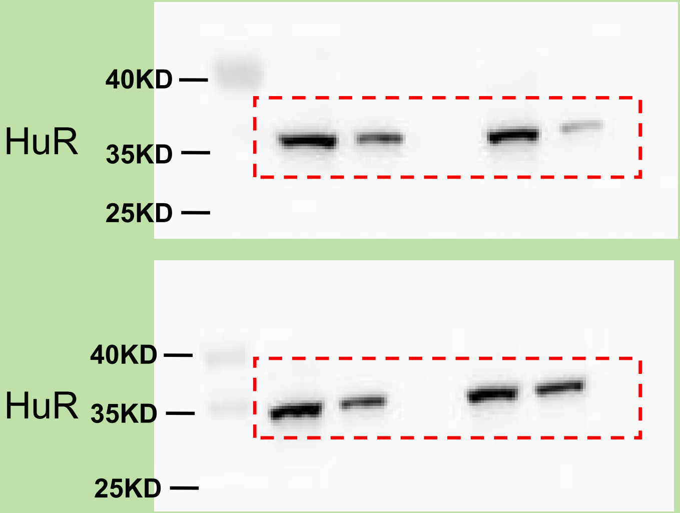

Fig 6N

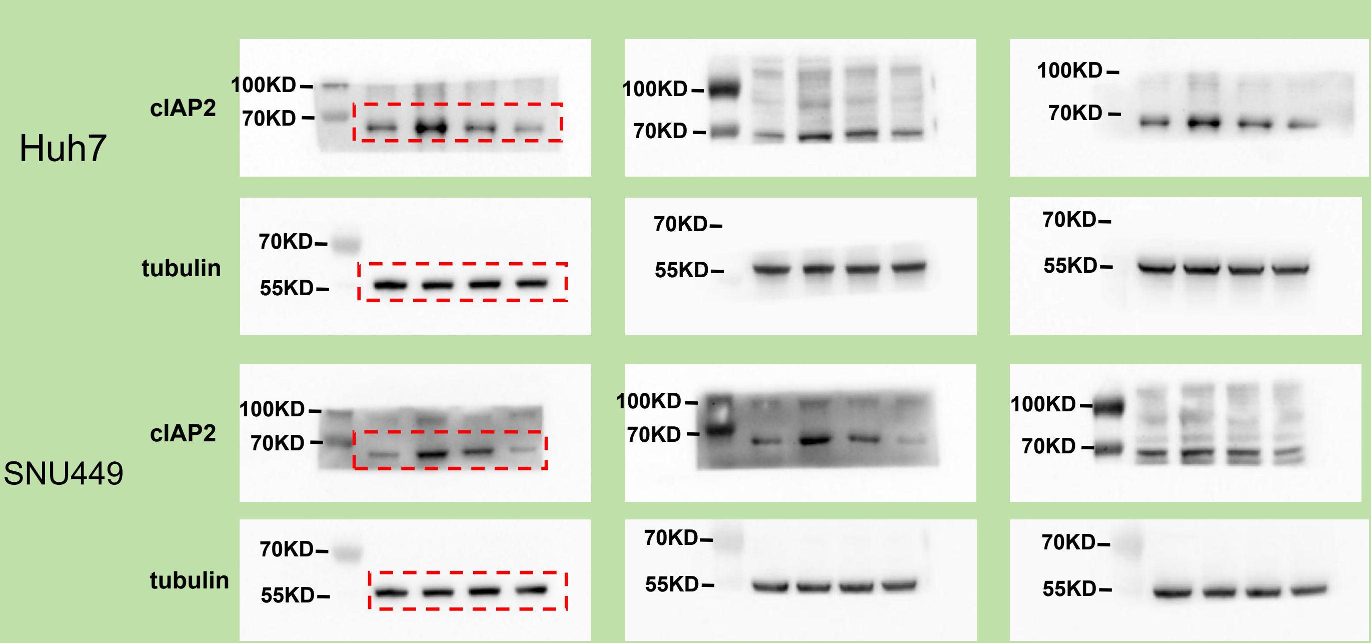

Fig 6P

Huh7

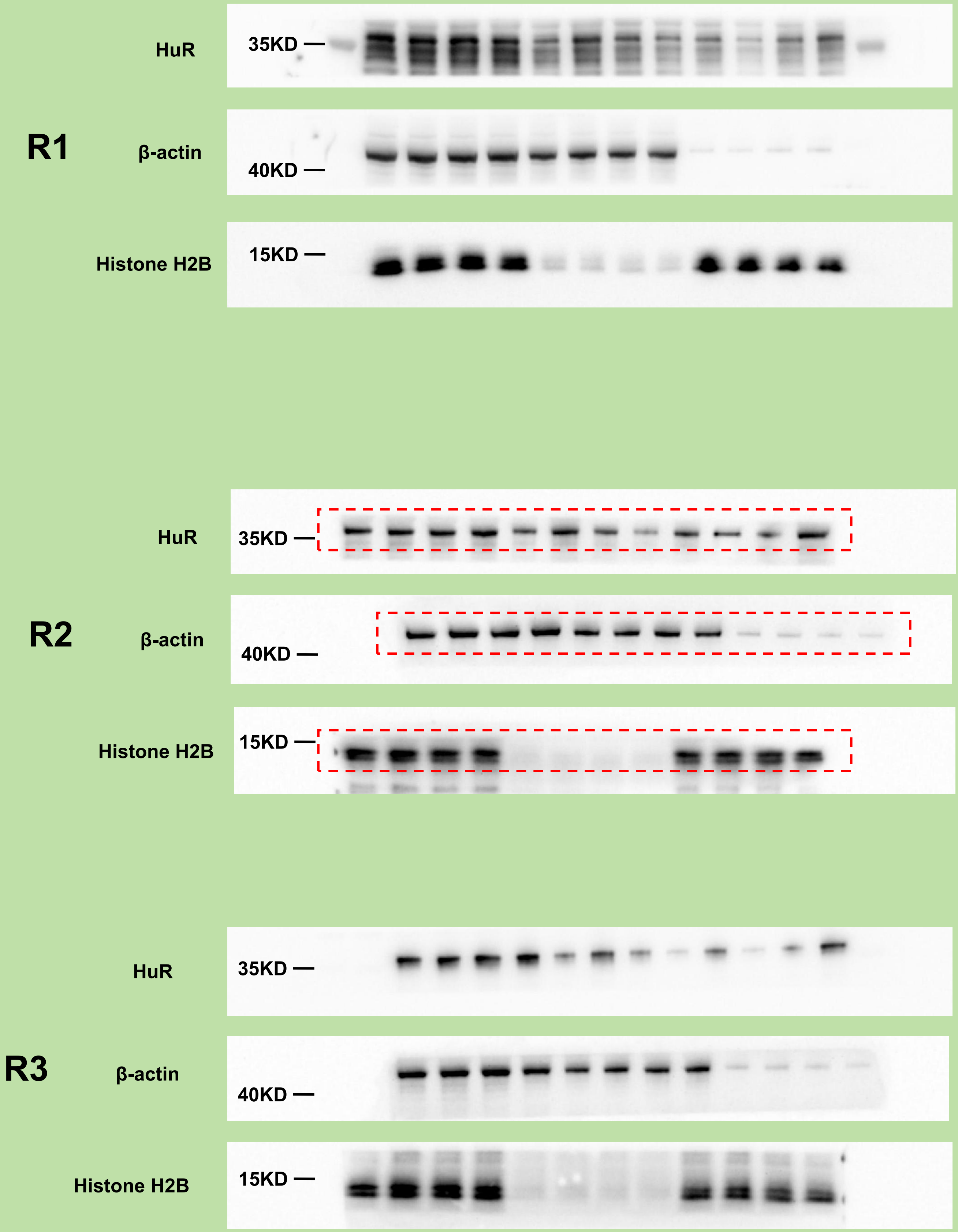
HuR
35KD
R1
β-actin
40KD
Histone H2B
15KD
HuR
35KD
R2
β-actin
40KD
Histone H2B
15KD
HuR
35KD
R3
β-actin
40KD
Histone H2B
15KD

Fig 6Q

SNU449

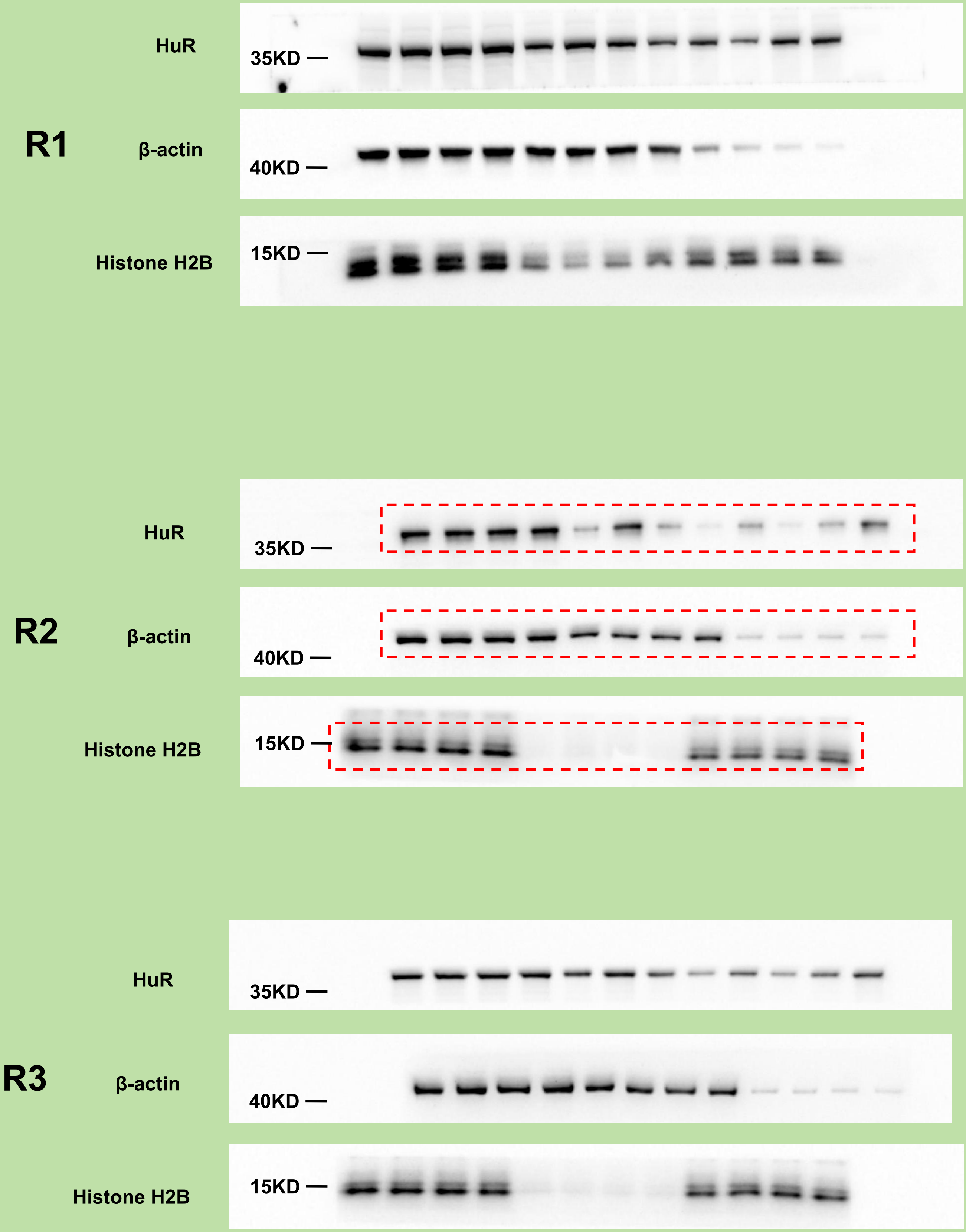

Supplement: Supplementary file 9 — original data [file 41419_2024_6570_MOESM9_ESM.pdf]
